# Dinitrogen complexes N_2_L_2_ (L = N_2_, CO, CS, NO^+^, CN^−^)

**DOI:** 10.1039/d5sc08399k

**Published:** 2026-02-09

**Authors:** Yahui Li, Chengxiang Ding, Lianbin Xie, Sudip Pan, Gernot Frenking

**Affiliations:** a Institute of Atomic and Molecular Physics, Jilin University Changchun 130023 China sudip@jlu.edu.cn; b Institute of Advanced Synthesis, School of Chemistry and Molecular Engineering, Nanjing Tech University Nanjing 211816 China frenking@chemie.uni-marburg.de; c Fachbereich Chemie, Philipps-Universität Marburg Hans-Meerwein-Strasse 4 D-35043 Marburg Germany; d Donostia International Physics Center (DIPC) M. de Lardizabal Pasealekua 3 20018 Donostia Euskadi Spain

## Abstract

Quantum chemical calculations using *ab initio* methods and density functional theory have been carried out on the equilibrium structures and the vibrational spectra of the (valence) isoelectronic compounds N_2_L_2_ (L = N_2_, CO, CS, NO^+^, CN^−^). The molecules have a *trans*-periplanar arrangement of the L_2_ ligands at the N_2_ unit. The complexes with L = N_2_, CO, NO^+^, CN^−^ are predicted as thermodynamically unstable for dissociation into N_2_ + 2L with Δ*G*^298^ value lying in between −257 kcal mol^−1^ (L = NO^+^) and −73 kcal mol^−1^ (L = CO), but the adduct N_2_(CS)_2_ is calculated as slightly stable with Δ*G*^298^ = 4 kcal mol^−1^. The homolytic dissociation reaction into two fragments N_2_L_2_ → 2 NL is energetically less favorable than the heterolytic fragmentation N_2_L_2_ → N_2_ + 2 L, which proceeds synchronously but asymmetrically. The activation barriers for the fragmentation reaction N_2_L_2_ → N_2_ + 2L have values between Δ*G*^≠^(298 K) = 17 kcal mol^−1^ for L = N_2_ and Δ*G*^≠^(298 K) = 84 kcal mol^−1^ for L = CS. The calculated vibrational frequencies suggest that the molecules N_2_L_2_ can be identified by the IR active antisymmetric stretching mode *ν*_as_ of the ligands L, which is blue shifted for L = CO (Δ = 55 cm^−1^) and L = NO^+^ (Δ = 118 cm^−1^) but it is red shifted for L = CS (Δ = −242 cm^−1^) and L = CN^−^ (Δ = −133 cm^−1^) relative to the *ν*_as_ mode of L = N_2_. The analysis of the bonding situation reveals that there is a total charge donation L→(^1^Γ-N_2_)←L in all complexes, ranging between 1.38 *e* (L = CN^−^) and 0.56 *e* (L = N_2_), except in the dication with L = NO^+^, where a small backdonation in reverse direction L←(^1^Γ-N_2_)→L with 0.10 *e* is calculated. EDA-NOCV calculations of N_6_ show that the best description of the bonding situation is given in terms of dative interactions N_2_→(^1^Γ-N_2_)←N_2_ between central N_2_ in the excited (1)^1^Γ_g_ singlet state and the terminal N_2_ fragments in the ^1^Σ_g_^+^ electronic ground state. In contrast, the best description of the complexes with L = CO, CS, NO^+^ is calculated for the interactions between the central N_2_ in the ^5^Σ_u_^+^ quintet state and the terminal ligands in the symmetry-adapted (L)_2_ quintet state. For N_2_L_2_ with L = CN^−^, it is found that the bonding is best described for the interaction between N_2_^−^ in the electronic quartet (^4^Σ_u_^+^) state and the terminal (L)_2_^−^ ligand as symmetry-adapted quartet. In contrast to the common bonding model for N_6_ using Lewis structures N^−^=N^+^=N–N=N^+^=N^−^, the donor–acceptor model N_2_→(N_2_)←N_2_ explains that the lowest activation barrier is found for the concerted cleavage of the two formal double bonds, leading to the experimentally observed dissociation into 3 N_2_.

## Abstract 2

Quantum chemical calculations using *ab initio* methods and density functional theory have been carried out on the equilibrium structures, energies and vibrational spectra of the (valence) isoelectronic compounds N_2_L_2_ (L = N_2_, CO, CS, NO^+^, CN^−^).

## Graphical abstract

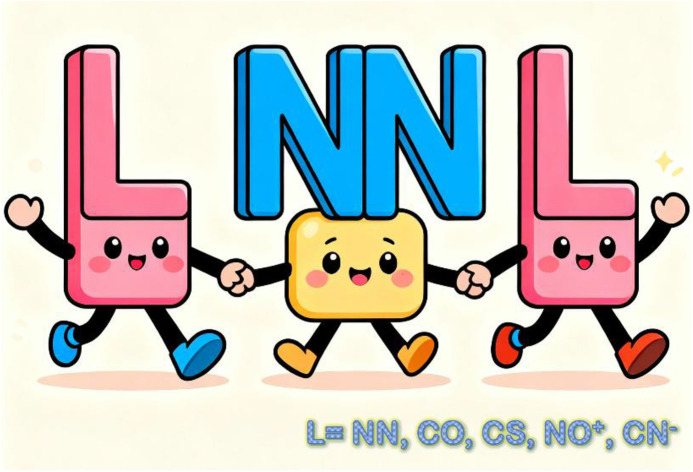

## Introduction

In 1964, Appel and Schöllhorn reported that triphenylphosphinazine, which was sketched with the formula Ph_3_P=N–N=PPh_3,_ is a thermally stable diamagnetic species that has a melting point of 184°.^1^ However, neither precise structural information about the compound was given in the work, nor in their further study.^2^ Later, quantum chemical calculations showed that N_2_(PPh_3_)_2_ is thermodynamically unstable for the release of N_2_ by ∼90 kcal mol^−1^,^3^ which raised doubts about the structure of the isolated compound. A subsequent X-ray structure analysis confirmed that the species is indeed triphenylphosphinazine, which has an antiperiplanar arrangement of the phosphine groups in Ph_3_P-(N_2_)-PPh_3_ with bending angles P–N–N of 107^o^, and a long N–N bond of 1.497 Å.^4^ The theoretical analysis of the bonding situation suggested that the N_2_ moiety binds through its highly excited (1)^1^Γ_g_ state, where the out-of-plane π and π* orbitals are doubly occupied and the in-plane π MO is vacant, which leads to strong dative interactions Ph_3_P→(N_2_)←PPh_3_ with N_2_ as a double Lewis acid. The same type of dative interactions was suggested for the related compound with NHC (N-Heterocyclic Carbene) ligand, NHC→(N_2_)←NHC, which was reported to have an antiperiplanar arrangement of the NHC groups and a long N–N bond of 1.415 Å.^5^

Very recently, Qian, Mardyukov and Schreiner (QMS) reported the synthesis, *via* gas-phase reaction, of the new nitrogen allotrope N_6_, which was trapped in low-temperature argon matrices at 10 K and as a film at liquid nitrogen temperature of 77 K.^6^ The molecule was identified by IR and UV-vis spectroscopy, and by *ab initio* calculations, which predict a structure where two N_3_ fragments are bonded in a *trans*-arrangement through a long (1.460 Å) N–N bond. The viewpoint of N_6_ as the dimer of N_3_ is reasonable, because hexanitrogen was synthesized by treating AgN_3_ with Cl_2_, which yields ClN_3_ that reacts with AgN_3_ and leads to the formation of the new nitrogen allotrope N_6_. Hexanitrogen was heralded as a molecule of the year 2025 wth a long N–N bond connecting two N_3_ fragments.^7^ But the structural similarity to the N_2_(PPh_3_)_2_ and N_2_(NHC)_2_ species led us suspect that the N_6_ species is another example of the compound class N_2_L_2_ where the ligands L are bonded through dative interactions L→(^1^Γ-N_2_)←L. Dinitrogen N_2_ is generally known as weakly bonded ligand, but the highly excited (1)^1^Γ_g_ state of the central N_2_ is a strong *σ* acceptor and strong π donor, which is capable to bind two N_2_ ligands. The (1)^1^Γ_g_ state of N_2_ is 294.3 kcal mol^−1^ above the X^1^Σ_g_^+^ ground state,^8^ which, however, does not occur in N_2_L_2_ as a free species, but as a reference state that is strongly stabilised by orbital interactions.

Another acceptor, which binds even eight N_2_ ligands in the octa-coordinated complexes M(N_2_)_8_ (M = Ca, Sr, Ba), is the alkaline-earth atom M in an excited triplet state with (n−1)d^2^ electron configuration *via* strong M→(N_2_)_8_ π backdonation.^9^

The new findings prompted us to investigate the electronic structure of N_6_ in terms of dative bonding N_2_→(^1^Γ-N_2_)←N_2_ and to compare the homolytic and heterolytic bond dissociation with quantum chemical methods. We also calculated the (valence) isoelectronic compounds N_2_L_2_ (L = N_2_, CO, CS, NO^+^, CN^−^). Here, we report about the equilibrium geometries, bond dissociation energies (BDEs), and the vibrational spectra of the molecules. We also present a thorough analysis of the nature of the chemical bonds using a variety of methods. The results may be useful as a guideline for future experimental studies.

## Methods

The geometrical optimizations, followed by the frequency calculations of N_2_L_2_ (L = N_2_, CO, CS, NO^+^, CN^−^) compounds, were first carried out at the M06-2X/cc-pVTZ level^10^ in their respective singlet and triplet spin states. Taking the most stable spin state (singlet), further reoptimizations and frequency calculations were performed at the CCSD(T)-Full/cc-pVTZ level.^11^ These calculations were done using the Gaussian 16 program.^12^ Natural atomic charges and Mayer bond order^13^ were calculated using NBO7 (ref. 14) and Multiwfn programs,^15^ respectively.

Energy decomposition analysis (EDA)^16^ in conjunction with the natural orbital for chemical valence theory (NOCV)^17^ was carried out at the M06-2X/TZ2P-ZORA^18^//CCSD(T)/cc-pVTZ level using the ADF 2020 package.^19^ The ZORA method considers relativistic effects, which are unimportant for this work, but further work by us on heavier analogues requires a uniform level of theory. In the EDA-NOCV^20^ analysis, the intrinsic interaction energy (Δ*E*_int_) between two fragments is dissected into three distinct energy components, as follows:

1 Δ*E*_int_ = Δ*E*_elstat_ + Δ*E*_Pauli_ + Δ*E*_orb_

The electrostatic Δ*E*_elstat_ term represents the quasiclassical electrostatic interaction between the unperturbed charge distributions of the prepared fragments. The Pauli repulsion, Δ*E*_Pauli_ accounts for the energy change during the transformation from the superposition of unperturbed electron densities of the individual fragments to a wavefunction that explicitly adheres to the Pauli principle, achieved through the necessary antisymmetrization and wavefunction renormalization. The orbital term Δ*E*_orb_ comes from the mixing of orbitals, charge transfer, and polarization between the isolated fragments.

The EDA-NOCV enables the partition of the total Δ*E*_orb_ into pairwise contributions of the orbital interactions that are very important to get a complete picture of the bonding. The charge deformation Δ*ρ*_k_(*r*), resulting from the mixing of the orbital pairs *ψ*_k_(*r*) and *ψ*_−k_(*r*) of the interacting fragments presents the amount and the shape of the charge flow due to the orbital interactions (eqn (2)), and the associated energy term Δ*E*_orb_ provides with the size of stabilizing orbital energy originated from such interaction (eqn (3)).2
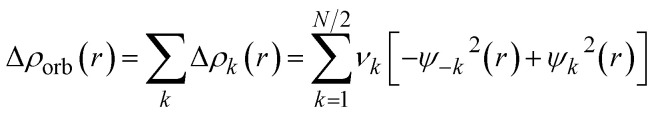
3
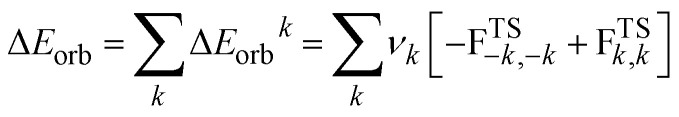


Several papers extensively discussed details of the EDA-NOCV method and its application, offering different perspectives and viewpoints.^21^

## Results and discussion

**Fig. 1:**
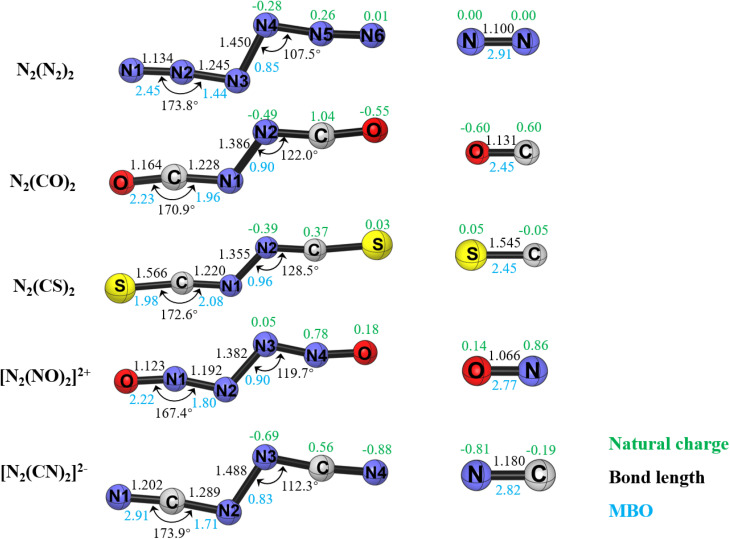
Minimum energy geometries, partial charges (*q*) and bond orders (*P*) of the N_2_L_2_ (L = N_2_, CO, CS, CN^−^, NO^+^) compounds and the ligands L at the CCSD(T)/cc-pVTZ level. The bond lengths are given in Å, bond angles in degree. All molecules have a C_2h_ point group and ^1^A_g_ electronic state.

Fig. 1 shows the calculated geometries, atomic partial charges (*q*), and bond orders (*P*) of the compounds N_2_L_2_ (L = N_2_, CO, CS, NO^+^, CN^−^) at the CCSD(T)/cc-pVTZ level. All molecules exhibit a *trans*-periplanar arrangement of the L_2_ ligands at the N_2_ unit with a C_2h_ point group and ^1^A_g_ electronic state. The singlet-triplet gap computed at the M06-2X/cc-pVTZ level is very high (39.7–65.7 kcal mol; see Table S1 in SI). The central N–N bond lengths in N_2_(N_2_)_2_ (1.450 Å) and [N_2_(CN)_2_]^2−^ (1.488 Å) compare favorably with the bond length in H_2_N–NH_2_ (1.445 Å),^6^ whereas the N–N bonds in N_2_(CO)_2_ (1.386 Å), N_2_(CS)_2_ (1.355 Å) and [N_2_(NO)_2_]^2+^ (1.382 Å) are significantly shorter. The small differences between our values for N_6_ and the data reported by QMS are due to the fact that the CCSD(T)/cc-pVTZ calculations were carried out by us with the full core option, whereas the previous study used the frozen core approximation. Geometry optimisations of a *syn* isomer did not result in another conformational isomer, but rather in the *trans* form as the only acyclic energy minimum structure. An extensive search of the potential energy surface using various starting geometries gave planar four-membered cyclic structures N_2_(CX)_2_ (X = O, S) as energy minima, which are > 25 kcal mol^−1^ less stable than the acyclic N_2_L_2_ forms. We also located nonplanar four-membered cyclic isomers for all species except for N_6_ where the central N–N bond is broken, which are > 40 kcal mol^−1^ higher in energy than the acyclic N_2_L_2_ forms. They are shown in Fig. S1 of the SI. Since the present work focuses on the dinitrogen complexes N_2_L_2_, we do not discuss the cyclic isomers in this work.

Table 1 gives the calculated BDEs for the fragmentation N_2_L_2_ → N_2_ + 2L at the CCSD(T)/cc-pVTZ level, which show a remarkably high variation. The purely electronic values suggest that [N_2_(NO)_2_]^2+^ is even less stable (*D*_e_ = −233.3 kcal mol^−1^) than N_2_(N)_2_ (*D*_e_ = −179.1 kcal mol^−1^). The exoenergetic values are much lower for [N_2_(CN)_2_]^2−^ (*D*_e_ = −116.7 kcal mol^−1^) and N_2_(CO)_2_ (*D*_e_ = −49.5 kcal mol^−1^), and N_2_(CS)_2_ is even predicted to be energetically stable (*D*_e_ = 27.7 kcal mol^−1^). The corrections by vibrational frequencies and entropic and thermal contributions lead to Δ*G*^298^ values where the N_2_L_2_ compounds are seen as thermodynamically unstable in the order L = NO^+^ > N_2_ > CN^−^ > CO, but the molecule N_2_(CS)_2_ is calculated as thermodynamically stable at room temperature with Δ*G*^298^ = 3.6 kcal mol^−1^.

**Table 1:** Computed bond dissociation energies *D*_e_, zero-point energies corrected bond dissociation energies *D*_0_, enthalpy change Δ*H* and free energy change Δ*G* of the processes (a) N_2_L_2_ → N_2_ + 2L and (b) N_2_L_2_ → 2NL at the CCSD(T)/cc-pVTZ level. All values are given in kcal mol^−1^

Complex	*D*_e_	*D*_0_	Δ*H*	Δ*G*
**(a) N**_**2**_ **L**_**2**_ **→ N**_**2**_ **+ 2L**				
N_2_(N_2_)_2_	−179.1	−184.1	−182.0	−200.7
N_2_(CO)_2_	−49.5	−55.8	−53.7	−73.4
N_2_(CS)_2_	27.7	21.8	23.6	3.6
[N_2_(CN)_2_]^2−^	−116.7	−122.2	−120.1	−139.7
[N_2_(NO)_2_]^2+^	−233.3	−238.7	−236.5	−256.8

**(b) N**_**2**_ **L**_**2**_ **→ 2NL**				
N_2_(N_2_)_2_	34.4	29.6	30.5	22.2
N_2_(CO)_2_	62.1	58.5	59.1	48.9
N_2_(CS)_2_	27.8	24.1	25.0	13.4
[N_2_(CN)_2_]^2−^	−51.8	−56.8	−55.8	−60.8
[N_2_(NO)_2_]^2+^	−70.2	−74.5	−73.7	−84.6

Table 1 also gives the BDEs for breaking the central N–N bond in the fragmentation N_2_L_2_ → 2 NL at the CCSD(T)/cc-pVTZ level. It becomes obvious that the homolytic bond rupture of the N–N bond is thermodynamically strongly disfavoured compared with the heterolytic cleavage of the L–N_2_–L bonds, except for L = CS. The energies of the two fragmentation reactions of the latter species are very similar, but the free energy of the heterolytic process (Δ*G* = 3.6 kcal mol^−1^) makes it clearly more favourable than the homolytic fragmentation (Δ*G* = 13.4 kcal mol^−1^). The calculated BDEs at the M06-2X/cc-pVTZ level are very similar (Table S2 of SI) to the CCSD(T)/cc-pVTZ values, which indicates that the DFT values are quite reliable.

**Fig. 2:**
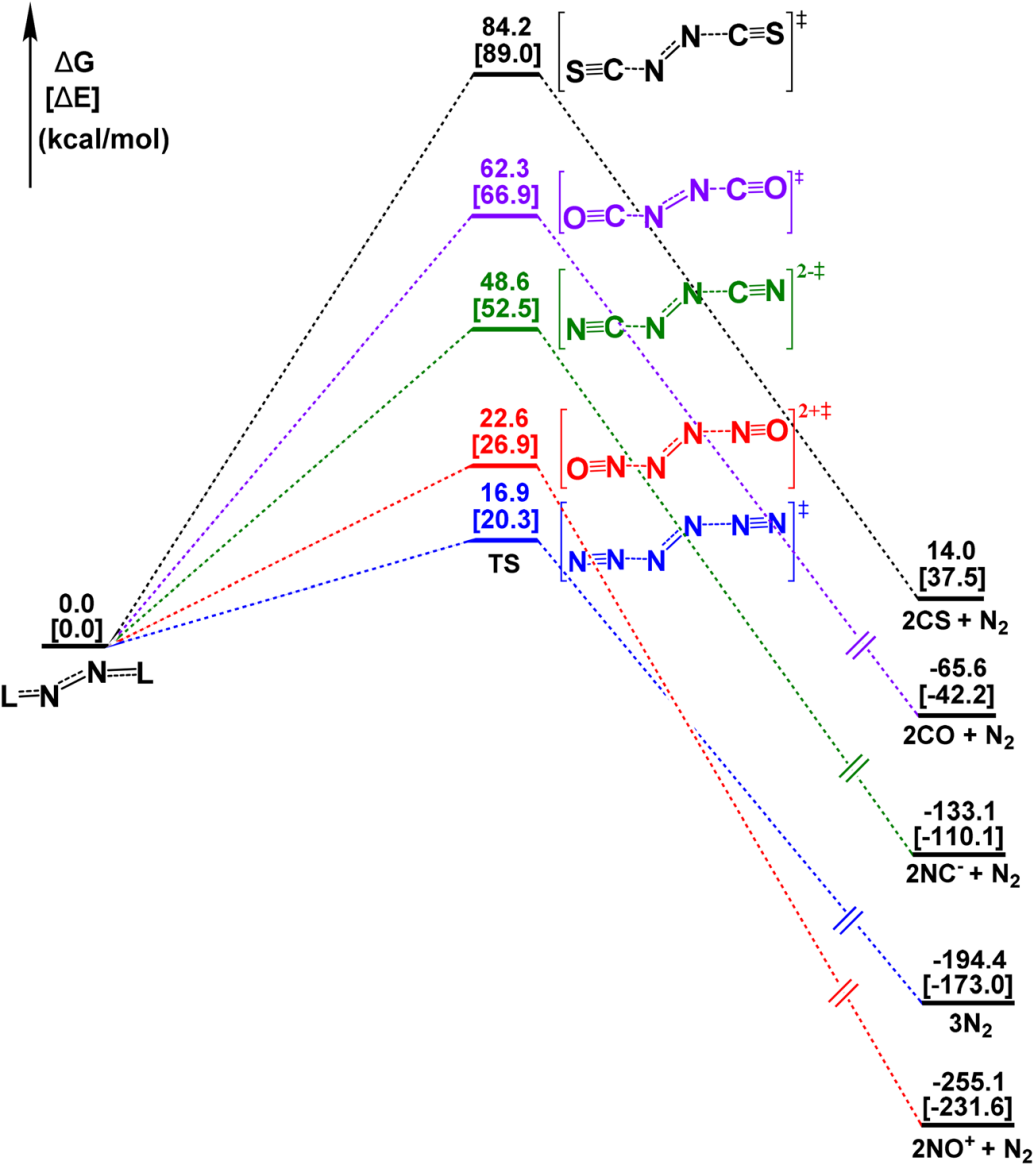
Energy profile for the reaction N_2_L_2_ → N_2_ + 2L at the CCSD(T)/cc-pVTZ//M06-2X/cc-pVTZ level.

Fig. 2 shows the reaction profile for the heterolytic dissociation reaction N_2_L_2_ → N_2_ + 2 L along with the calculated activation free energy barriers Δ*G*^≠^(298 K), which vary between 16.9 kcal mol^−1^ (L = N_2_) and 84.2 kcal mol^−1^ (L = CS). The trend of the Δ*G*^≠^ follows, in general, the reaction energies, except for the very exergonic reactions where L = N_2_, NO^+^. The calculated barrier for the fragmentation of N_2_ is in good agreement with the value reported by QMS (14.8 kcal mol^−1^).^6^ The computed value of the *T*_1_ diagnostics suggests that the multi-reference character of the CCSD(T) calculations of the N_2_L_2_ complexes and the transition states is very low (Table S3, SI), which indicates that the single-reference approach is sufficient. The activation barrier for the homolytic reaction N_6_ → 2 N_3_ was reported by QMS to be significantly higher (Δ*G*^≠^(298 K) = 26.1 kcal mol^−1^) than the heterolytic process.^6^ We carried out energy calculations with stretched N–N distances of the other L_2_L_2_ species, which suggest that the barriers are also higher than for the heterolytic fragmentation. Since the NL fragments are also less stable than N_2_ + 2 L (Table 1), it is unlikely that the homolytic reaction course plays a role in the fragmentation reaction of the N_2_L_2_ species.

The higher barrier for breaking the bond in the central N_2_ moiety of N_6_ compared to breaking the (N_2_)–(N_2_)_2_ bonds is surprising, given the calculated bond orders, which are much higher and have shorter distances in the latter bonds than the former one. It turns out that the energy required to break a bond depends not only on the strength of the bond, but also on the reorganisation of the electronic structure of the fragments during the cleavage reaction. The N_3_–N_3_ bond in N_6_ is weaker than the (N_2_)–(N_2_)_2_ bonds, but the electronic charge migration during rupture of the latter heterolytic process is energetically more favourable than the homolytic reaction. The lower barrier for breaking the (N_2_)–(N_2_)_2_ bonds than the N_3_–N_3_ bond supports the use of our bonding model for N_6_ in terms of dative interactions N_2_→(N_2_)←N_2_ rather than the more conventional model using Lewis structures N^−^=N^+^=N–N=N^+^=N^−^, since it accounts for the experimental finding that N_6_ directly dissociated into 3 N_2_. The same applies for N_2_(CO)_2_, which photolytically dissociates into N_2_ + 2 CO whereas cleavage into NCO radical was observed only to a small extent.^22*a*^

**Fig. 3:**
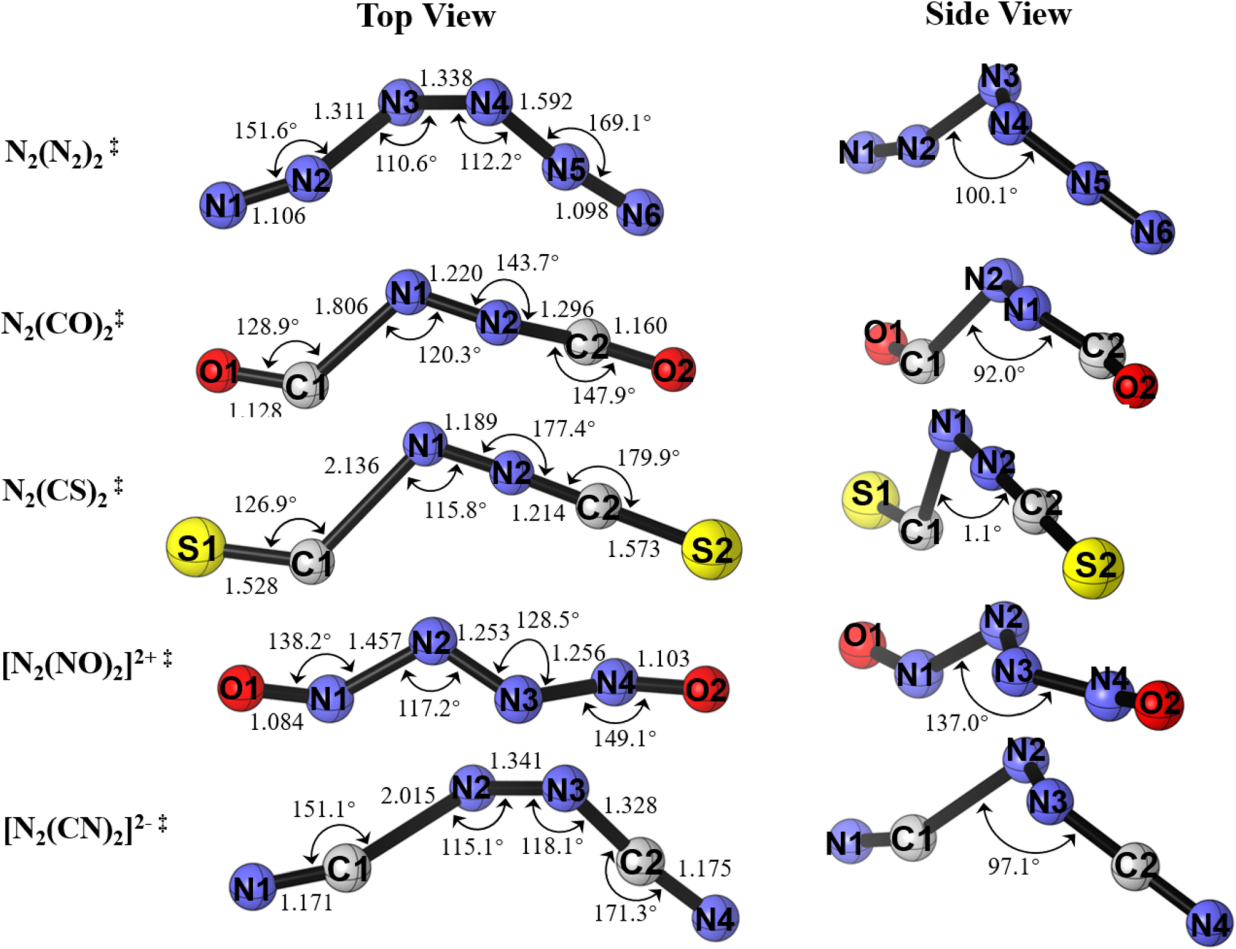
Geometries of the transition states of N_2_L_2_ (L = N_2_, CO, CS, CN^−^, NO^+^) compounds for the dissociation into 2L and N_2_ at the M06-2X/cc-pVTZ level. The top view gives the bond lengths and bond angles, and the side view gives the dihedral angles L-N_2_-L. The bond lengths are given in Å and the angles in degree.

Examination of the transition state structures for the dissociation reaction reveals surprising features (Fig. 3). The geometries of [N_2_L_2_]^≠^ possess a non-planar staggered geometry with a *syn* conformation of the ligands except for [N_2_(CS)_2_]^≠^, which exhibits a nearly linear NNCS moiety with one CS ligand, where the bond to the second CS ligand is significantly stretched. This implies that the fragmentation reaction may possibly be a two-step process in which an intermediate product, NN-L is formed along the dissociation reaction. We calculated the intrinsic reaction coordinate starting from the transition states and found that it smoothly connects to the reaction products N_2_ + 2L. The fragmentation reaction is thus predicted as a concerted but highly asynchronous process where the two N_2_–L bonds break one after the other. Note that the central N–N bond of the transition state [N_2_L_2_]^≠^ is clearly shorter than in the equilibrium structures.

**Fig. 4:**
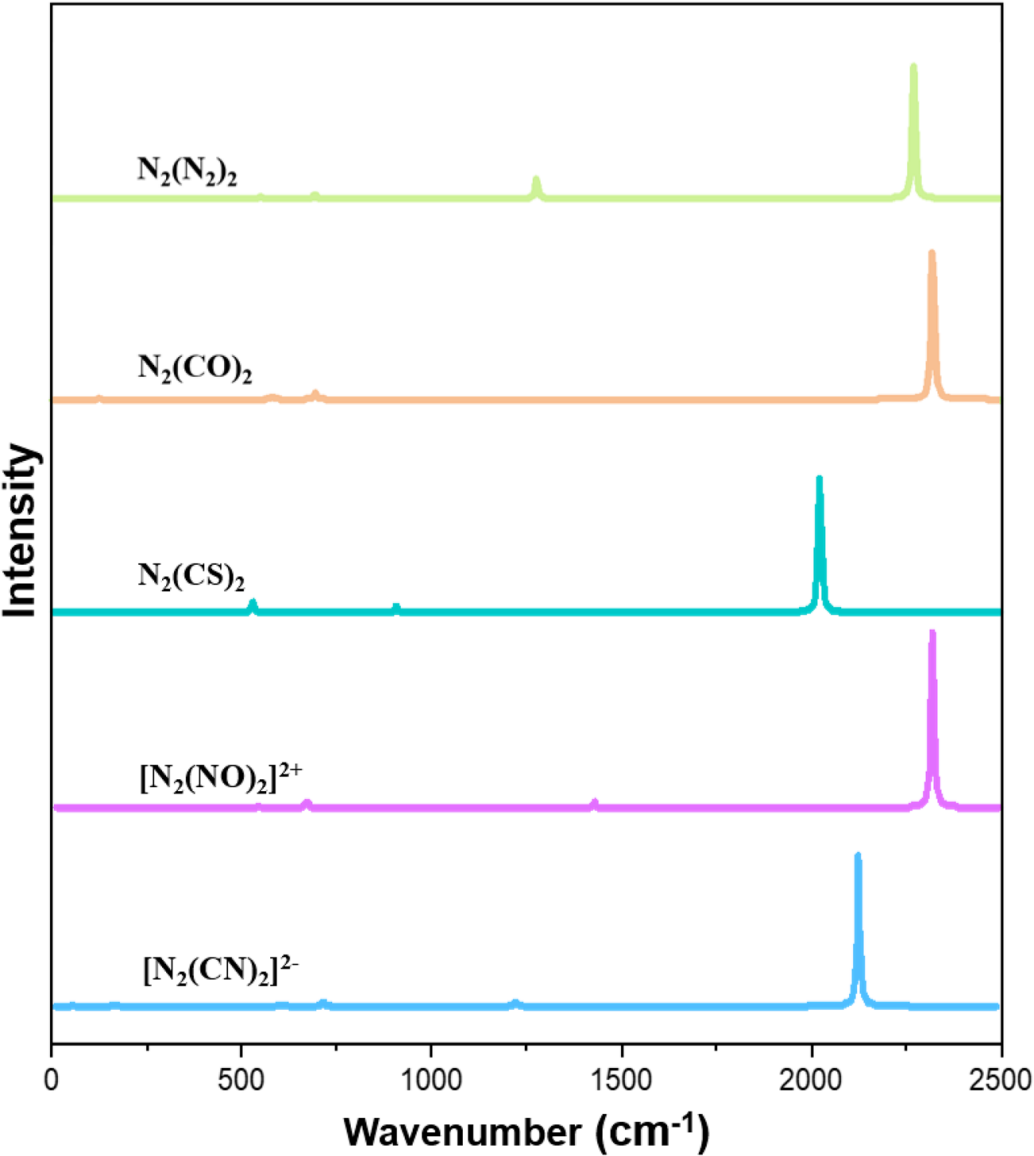
Calculated IR spectra of N_2_L_2_ complexes (L = N_2_, CO, CS, CN^−^, NO^+^) at the M06-2X/cc-pVTZ level.

Hexanitrogen was clearly identified by spectroscopic signals, and the authors presented a careful analysis of the vibrational spectrum of the molecule.^6^ The IR spectrum of N_6_ exhibits an intense vibrational band at 2076.6 cm^−1^, which comes from an asymmetric stretching mode of the terminal N_2_ ligands. Fig. 4 shows the calculated IR spectra of the five molecules N_2_L_2_. They show a similar pattern where the asymmetric stretching mode *ν*_as_ of the terminal L_2_ ligands, which has the second largest wavenumber of all 12 fundamentals, exhibits the strongest signal. The symmetric stretching mode *ν*_s_ with a slightly higher wavenumber is IR inactive. The calculated wavenumber of 2263.7 cm^−1^ for the asymmetric mode of N_6_ is blue shifted for L = CO (Δ = 55 cm^−1^) and L = NO^+^ (Δ = 118 cm^−1^), but it is red shifted for L = CS (Δ = −242 cm^−1^) and L = CN^−^ (Δ = −133 cm^−1^). The calculated frequency shift of the intense asymmetric stretching mode *ν*_s_ is very helpful information for identifying the molecules.

In addition to N_6_ [N_2_(N_2_)_2_], the complexes N_2_(CO)_2_ and N_2_(CS)_2_ have been synthesised and identified spectroscopically and they were also the subject of theoretical works. The dicarbonyl adduct was introduced as diisocynate O=C=N–N=C=O following the conventional descriptions using Lewis structures.^22^ Photolysis of the matrix-isolated N_2_(CO)_2_ gives N_2_ + 2 CO as products, which agrees with our calculated reaction profile (Fig. 2). The IR spectrum shows one very intensive band at 2200.6 cm^−1^ for the asymmetric NCO stretching mode besides several signals with lower intensity.^22*a*^ The comparison with the IR spectrum of N_6_ gives a red shift of 124 cm^−1^, which agrees with the direction but is higher than our computed value of 54 cm^−1^. The diisothiocyanate complex N_2_(CS)_2_ was identified as product of the photolysis of the energetically lower lying isomer S_2_(CN)_2_ with a characteristic vibrational mode of the IR spectrum at 1910 cm^−1^.^23^ The experimental red-shift compared to the asymmetric stretching mode of N_6_ is also given by our calculations, but the observed value of −166 cm^−1^ is smaller than our computed value of −242 cm^−1^. The computed wavenumber for [N_2_(NO)_2_]^2+^ (2382 cm^−1^) agrees quite well with the experimental value of NO^+^ (2340 cm^−1^) whereas the calculated value for [N_2_(CN)_2_]^2−^ (2131 cm^−1^) is red-shifted compared with an approximately interaction-free anion CN^−^ (2244 cm^−1^).^24^ The complete set of the calculated vibrational spectra of the five molecules N_2_L_2_ is given in Table S4 of the SI.

We analyzed the bonding situation in N_2_L_2_ with a variety of methods. Fig. 1 shows that the Mayer bond order (MBO) of the central N–N bond is between 0.83 (L = CN^−^) and 0.96 (L = CS), which is a rather small variation considering the differences in the N–N bond length between 1.488 Å (L = CN^−^) and 1.355 Å (L = CS). The atomic partial charges suggest that there is a total charge donation in the neutral complexes L→(N_2_)←L with 0.98 *e* (L = CO), 0.78 *e* (L = CS), and 0.56 *e* (L = N_2_). There is an even stronger charge donation of 1.38 *e* in the dianion (L = CN^−^) and a small backdonation in reverse direction L←(N_2_)→L with 0.10 *e* in the dication (L = NO^+^).

**Fig. 5:**
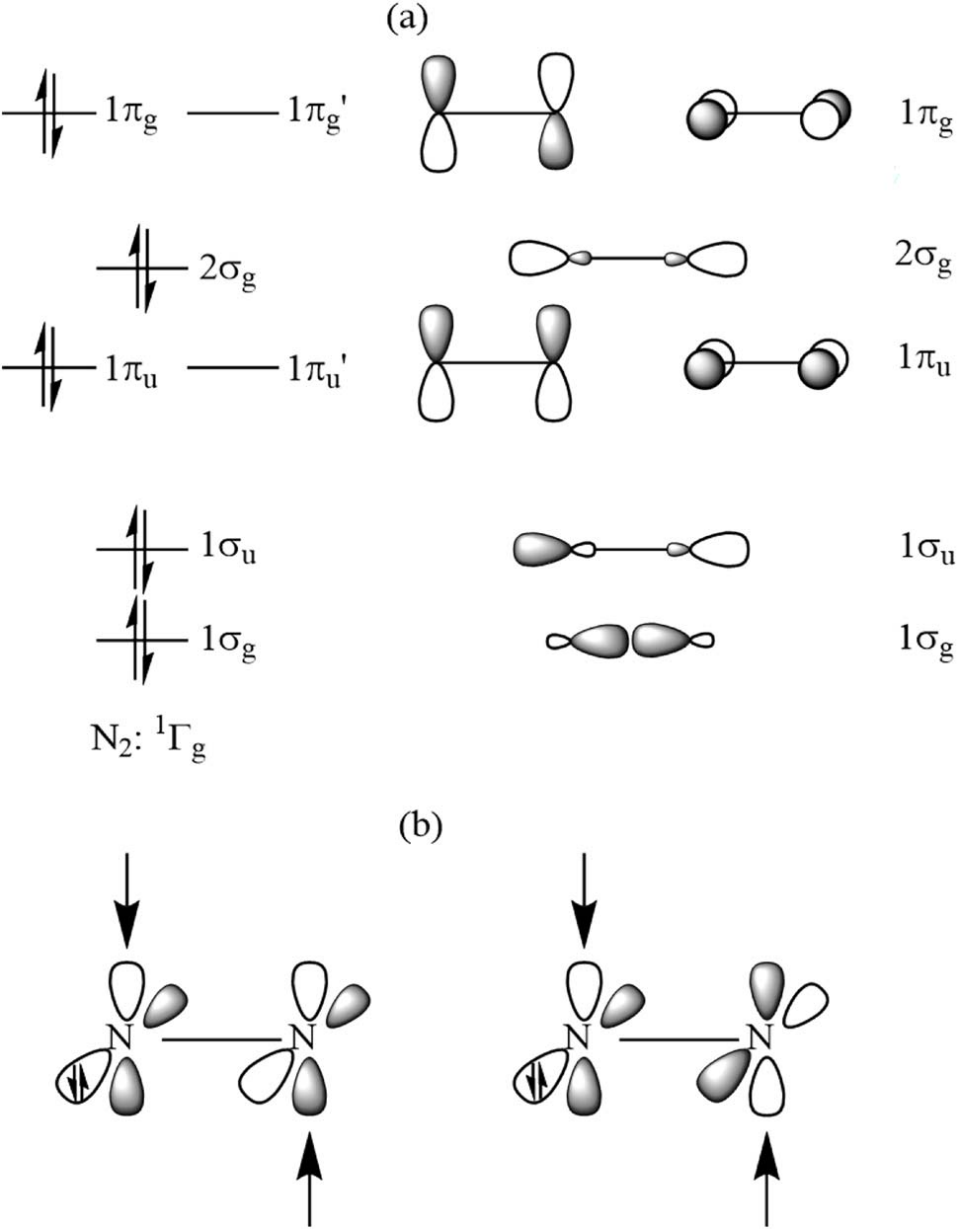
(a) Schematic representation of the (1)^1^Γ_g_ state of N_2_ and the associated orbitals. (b) Donation of the plus and minus combination of the lone-pair donor orbitals of L into the vacant in-plane π and π* orbitals of N_2_. The orbital numbering refers to the valence orbitals of N_2_.

We proposed in our earlier study that the diatomic N_2_ species in N_2_(PPh_3_)_2_ binds through its highly excited (1)^1^Γ_g_ state where the out-of-plane π and π* orbitals are doubly occupied.^4^Fig. 5 shows schematically the valence MOs of N_2_ in the (1)^1^Γ_g_ state. The donation of the L_2_ ligands L→(N_2_)←L may take place into the vacant 1π_u_′ (bonding) and 1π_g_′ (antibonding) orbitals of N_2_. Note that the assignment of π symmetry refers to free N_2_, which has two mirror planes that contain the atoms. The complexes L-N_2_-L have only one mirror plane and the donation is correctly assigned as in-plane σ(+,−) and σ(+,+) orbital interaction.

**Fig. 6:**
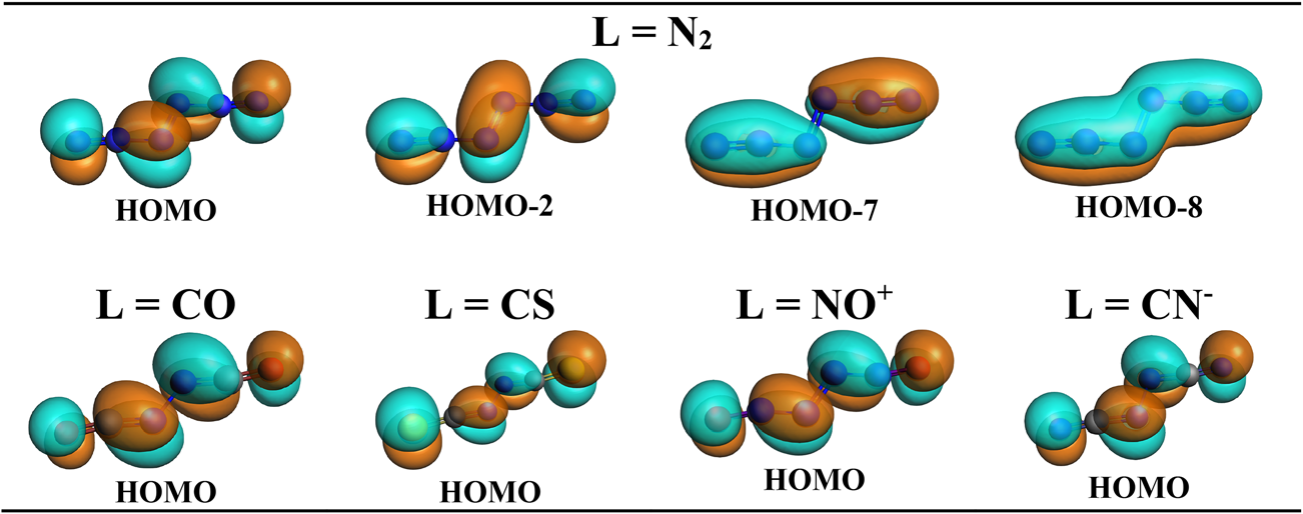
Top row: plot of the four occupied valence π MOs of N_6_. Bottom row: plot of the HOMO of N_2_L_2_ with L = CO, CS, NO^+^, CN^−^.

There are two occupied out-of-plane π MOs, 1π_u_ (bonding) and 1π_g_ (antibonding), in the (1)^1^Γ_g_ state of N_2_. If this holds true also for the N_2_L_2_ complexes in the present study, the number of occupied valence π MOs should be four (π and π* from N_2_ and one π from each ligand L) and not three. Examination of the shape of the Kohn–Sham MOs shows that this is indeed the case. Fig. 6 displays the four occupied valence π MOs of N_6_. The number of nodes follows the common symmetry rules with zero (HOMO-8), one (HOMO-7), two (HOMO-2), and three (HOMO). The same number of occupied valence π is found for the other N_2_L_2_ complexes. The complete set of occupied valence MOs of all N_2_L_2_ complexes is presented in Fig. S2–S6. Fig. 6 shows also the HOMO of the N_2_L_2_ complexes, which can be identified with the out-of-plane π* orbital of N_2_ mixing with the antibonding π* orbitals of the ligands L.

A more detailed insight into the nature of the N_2_–L_2_ bonds is provided by the EDA-NOCV analysis of the interactions between the fragments. In our previous study of N_2_(PPh_3_)_2_, we considered only neutral N_2_ in the excited (1)^1^Γ_g_ state and 2 PPh_3_ ligands in the electronic ground state.^4^ We analyzed N_2_L_2_ using various electronic states and charges of the fragments N_2_ and 2L. Numerous studies have shown that the strength of the orbital term Δ*E*_orb_, which considers the change in the wave function during bond formation, is a reliable indicator of the optimal fragments for describing the bond between them.^7,25^ The fragments with the lowest absolute values of Δ*E*_orb_ are the best choice for explaining the bonding interactions. Table 2 shows the numerical results for L = N_2_.

**Table 2:** EDA-NOCV results of the complex N_2_L_2_ (L = N_2_) considering L_2_ as one fragment and central N_2_ as another fragment at the M06-2X/TZ2P-ZORA//CCSD(T)/cc-pVTZ level. Energy values are given in kcal mol^−1^

Energies	Orbital interaction	N_2_ (singlet) + 2L (singlet)	N_2_ (triplet) + 2L (triplet)	N_2_ (quintet) + 2L (quintet)	N_2_^−^(doublet) + 2L^+^(doublet)	N_2_^−^(quartet) + 2L^+^(quartet)
Δ*E*_int_		−165.0	−368.7	−401.3	−387.3	−519.0
Δ*E*_Pauli_		1036.0	1022.8	1028.3	1042.8	1090.1
Δ*E*_elstat_^a^		−364.9 (30.4%)	−388.8 (27.9%)	−438.1 (30.6%)	−538.1 (37.6%)	−615.1 (38.6%)
Δ*E*_orb_^a^		−836.1 (69.6%)	−1002.7 (72.1%)	−991.5 (69.4%)	−894.5 (62.4%)	−980.0 (61.4%)
Δ*E*_orb(1)_^b^	L–NN–L σ-bond (+,−)	−328.7 (39.3%)	−317.3 (31.6%)	−328.7 (33.2%)	−329.8 (36.9%)	−350.4 (35.8%)
Δ*E*_orb(2)_^b^	L–NN–L σ-bond (+,+)	−296.4 (35.5%)	−296.9 (29.6%)	−314.1 (31.7%)	−261.4 (29.2%)	−337.7 (34.5%)
Δ*E*_orb(3)_^b^	L–NN–L π-bond (+,−)	−98.6 (11.8%)	−174.9 (17.4%)	−151.4 (15.3%)	−123.1 (13.8%)	−90.9 (9.3%)
Δ*E*_orb(4)_^b^	L–NN–L π-bond (+,+)	−44.7 (5.3%)	−86.5 (8.6%)	−94.8 (9.6%)	−54.0 (6.0%)	−56.2 (5.7%)
Δ*E*_orb(rest)_^b^		−67.7 (8.1%)	−127.1 (12.7%)	−102.2 (10.3%)	−126.2 (14.1%)	−144.8 (14.8%)

a The values in parentheses give the percentage contribution to the total attractive interactions Δ*E*_elstat_ + Δ*E*_orb_.

b The values in parentheses give the percentage contribution to the total orbital interactions Δ*E*_orb_.

It becomes obvious that the chemical bonds in N_6_ between the central N_2_ moiety and the terminal N_2_ species are indeed best described in terms of dative interactions N_2_→(N_2_)←N_2_ between central N_2_ in the excited (1)^1^Γ_g_ singlet state and two terminal N_2_ fragments in the ^1^Σ_g_^+^ electronic ground state. The interactions between central N_2_ in the ^3^Σ_u_^+^ triplet state and the terminal ligands in the symmetry-adapted (N_2_)_2_ triplet state, which exhibit a mixture of electron-sharing and dative bonds, give a bigger Δ*E*_orb_ value. The same holds for the formation of electron-sharing double bonds between central N_2_ in the ^5^Σ_u_^+^ quintet state and the terminal ligands in the symmetry-adapted (N_2_)_2_ quintet state. EDA-NOCV calculations using central N_2_^−^ as an anion in the electronic doublet (^2^Σ_g_^+^) or quartet (^4^Σ_u_^+^) state and the terminal (N_2_)_2_^+^ ligand as a cation in the symmetry-adapted doublet or quartet state also result in bigger Δ*E*_orb_ values.

**Fig. 7:**
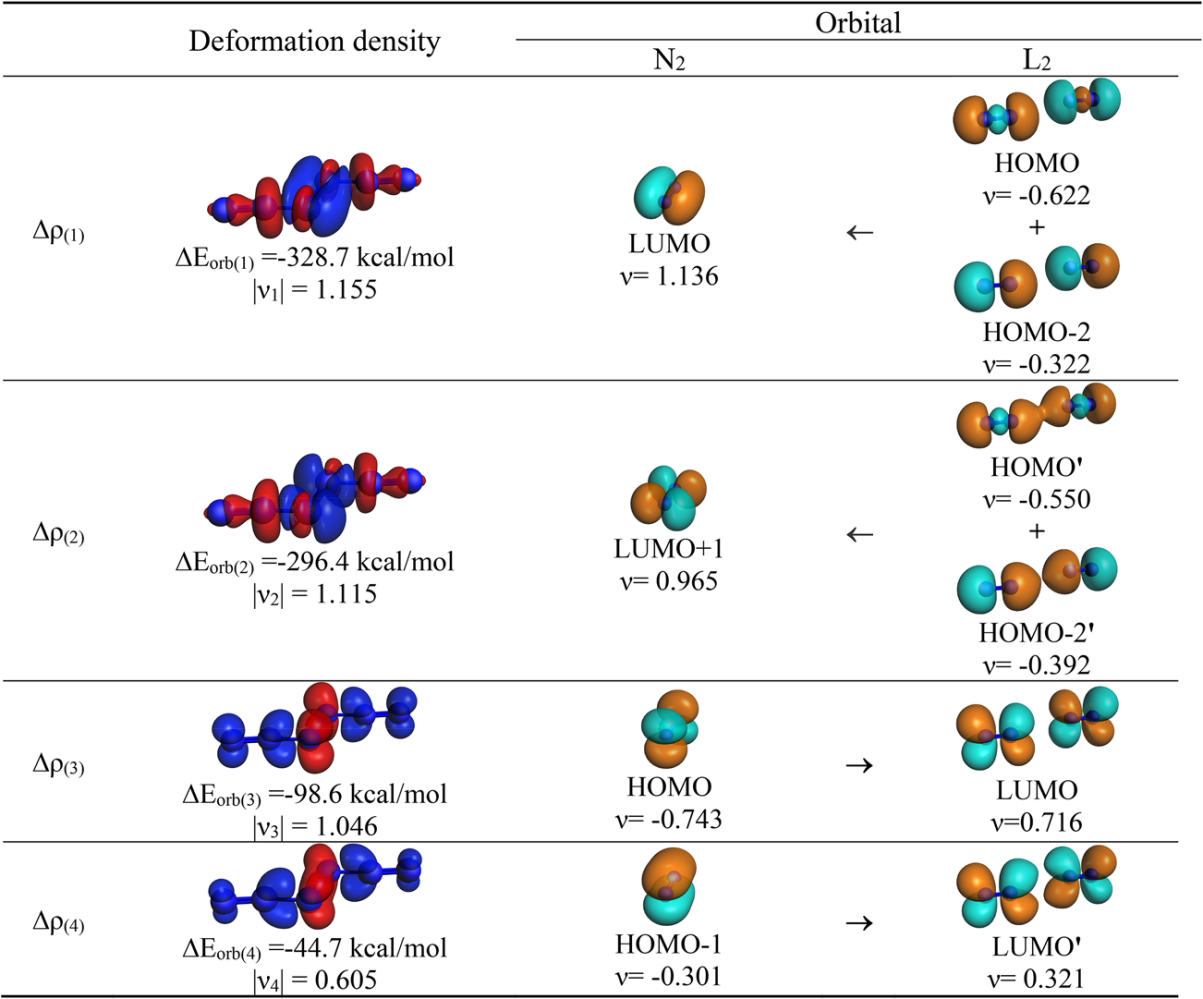
Plot of the deformation densities, Δ*ρ*_(1–4)_ shown as the electronic charge corresponding to Δ*E*_orb(1–4)_ and the related interacting orbitals of N_2_L_2_ (L = N_2_) at the M06-2X/TZ2P-ZORA//CCSD(T)/cc-pVTZ level using NN (^1^Γ_g_) + L_2_ (singlet) as interacting fragments. The eigenvalues *ν* indicate the size of the charge flow. The direction of charge flow is red → blue. The isovalue for Δ*ρ*_(1–4)_ is 0.003 au.

Further examination of the dative interactions between central N_2_ and the terminal N_2_ ligands reveals that they come mainly from N_2_→(N_2_) N_2_*σ* donation through out-of-phase (Δ*E*_orb(1)_) and in-phase (Δ*E*_orb(2)_) orbital pairs, which provide 75% of the total orbital (covalent) bonding. The π backdonation N_2_←(N_2_)→N_2_*via* out-of-phase (Δ*E*_orb(3)_) and in-phase (Δ*E*_orb(4)_) orbital interactions deliver only 17% of Δ*E*_orb_. The nature of the individual orbital interactions Δ*E*_orb(1)_–Δ*E*_orb(4)_ becomes clear when considering the corresponding deformation densities and the associated orbitals, which are shown in Fig. 7. The dominant orbital interactions through (+,−) and (+,+) *σ* donation N_2_→(N_2_)←N_2_ nicely explain the bond shortening of the central N_2_ moiety compared to free N_2_ in the ^1^Γ state, which has a calculated bond length of 1.608 Å and is 294.3 kcal mol^−1^ above the X^1^Σ_g_^+^ ground state.^8^ The donation takes place into the vacant in-plane and out-of-plane π orbitals of N_2_, which are bonding orbitals (see Fig. 5). Note that the (1)^1^Γ_g_ state of N_2_ is a reference state which is strongly stabilized by the orbital interaction. It is not formed as a free species during the reaction process.

The numerical EDA-NOCV results of the other N_2_L_2_ compounds are given in Tables S5–S8 of SI. In contrast to the results for N_6_, the lowest Δ*E*_orb_ values with L = CO, CS, NO^+^ are calculated for the interactions between the central N_2_ in the ^5^Σ_u_^+^ quintet state and the terminal ligands in the symmetry-adapted (L)_2_ quintet state, which indicate electron-sharing double bonds between the fragments. The central N–N bond in the latter species is much shorter (between 1.355–1.386 Å) than in N_6_ (1.450 Å). The lowest Δ*E*_orb_ value of N_2_L_2_ with L = CN^−^ is found for the interactions between N_2_^−^ in the electronic quartet (^4^Σ_u_^+^) state and the terminal (CN)_2_^−^ ligands as symmetry-adapted quartets. The individual orbital interactions are identified by the corresponding deformation densities and the associated orbitals, which are shown in Fig. S7–S14. The latter electron-sharing interactions also lead to four occupied π valence MOs in all five N_2_L_2_ molecules.

In order to compare the N_2_L_2_ compounds with each other, we have chosen the same fragments with the central N_2_ in the excited (1)^1^Γ_g_ singlet state and two terminal N_2_ fragments in the ^1^Σ_g_^+^ electronic ground state as model for the bonding interactions. Table 3 gives the numerical results. The calculated interaction energies Δ*E*_int_ show the same order CS > CO > CN^−^ > NO^+^ > N_2_ as the calculated activation barriers Δ*E*^≠^ (Fig. 2), which indicates that the choice of the singlet fragments is a valid model for the trend of the chemical bonds. The orbital (covalent) interactions always make the largest percentage contribution to the chemical bonds, which is particularly high for L = NO^+^ (75%). The breakdown of Δ*E*_orb_ into the pairwise orbital interactions reveals that the *σ* donations through out-of-phase (Δ*E*_orb(1)_) and in-phase (Δ*E*_orb(2)_) orbital pairs are always the largest components of the total orbital bonding. The percentage contribution of Δ*E*_orb(1)_ and Δ*E*_orb(2)_ is higher for L = CN^−^ and lower for L = NO^+^, which is due to the charges of the ligands. A surprising result concerns the low contribution of the π backdonation Δ*E*_orb(3)_ and Δ*E*_orb(4)_ for L = CO, which is smaller than for L = N_2_. CO is known to be a better π acceptor than N_2_ in transition metal complexes.^26^ The peculiar results for the orbital interactions in the neutral N_2_L_2_ compounds can be explained with the dominance of the *σ* donation L→N_2_←L over π backdonation L←N_2_→L, which comes to the fore by the calculated partial charges (Fig. 1). The energy of the σ lone-pair HOMO suggests that the donor strength of the ligands has the order CS (*ε* = −10.1 eV) > CO (*ε* = −12.2 eV) > N_2_ (*ε* = −13.9 eV). But the overall strength of the dative interactions cannot simply be derived from the orbital interactions. The orbital interaction Δ*E*_orb_ of the negatively charged CN^−^ is significantly weaker (−972.1 kcal mol^−1^) than that of NO^+^ (−1088.0 kcal mol^−1^), but the total interaction energy Δ*E*_int_ of the former ligand is clearly higher (−301.4 kcal mol^−1^) than the latter (−202.5 kcal mol^−1^). The electrostatic interaction Δ*E*_elstat_ but also the Pauli repulsion Δ*E*_Pauli_, which makes the largest contribution to Δ*E*_int_, are equally relevant for the trend of the interatomic interactions. It has been shown that the Pauli repulsion is the crucial factor for the equilibrium geometry of molecules.^27^

**Table 3:** EDA-NOCV results of complexes N_2_-L_2_ (L = N_2_, CO, CS, NO^+^, CN^−^) considering L_2_ as one fragment and central N_2_ as another fragment in the singlet states (^1^Γ_g_ state of N_2_ and symmetry adapted singlet state of L_2_) at the M06-2X/TZ2P-ZORA//CCSD(T)/cc-pVTZ level. Energy values are given in kcal mol^−1^

Energies	Orbital interaction	L = N_2_	L = CO	L = CS	L = NO^+^	L = CN^−^
Δ*E*_int_		−165.0	−306.0	−387.0	−202.5	−301.4
Δ*E*_Pauli_		1036.0	1400.4	1542.2	1242.3	1155.6
Δ*E*_elstat_^a^		−364.9 (30.4%)	−530.8 (31.1%)	−595.4 (30.9%)	−356.9 (24.7%)	−484.9 (33.3%)
Δ*E*_orb_^a^		−836.1 (69.6%)	−1175.7 (68.9%)	−1333.8 (69.1%)	−1088.0 (75.3%)	−972.1 (66.7%)
Δ*E*_orb(1)_^b^	L–NN–L in-plane(σ)-bond (+,−)	−328.7 (39.3%)	−483.0 (41.1%)	−536.4 (40.2%)	−373.2 (34.3%)	−405.8 (41.7%)
Δ*E*_orb(2)_^b^	L–NN–L in-plane(σ)-bond (+,+)	−296.4 (35.5%)	−434.3 (37.4%)	−493.4 (37.0%)	−322.3 (29.6%)	−400.0 (41.1%)
Δ*E*_orb(3)_^b^	L–NN–L out-of-plane(π)-bond (+,−)	−98.6 (11.8%)	−76.1 (6.5%)	−134.6 (10.1%)	−182.1 (16.7%)	−70.4 (7.2%)
Δ*E*_orb(4)_^b^	L–NN–L out-of-plane(π)-bond (+,+)	−44.7 (5.3%)	−45.2 (3.8%)	−48.0 (3.6%)	−75.5 (6.9%)	−34.6 (3.6%)
Δ*E*_orb(rest)_^b^		−67.7 (8.1%)	−137.1 (11.7%)	−121.4 (9.1%)	−134.9 (12.4%)	−61.3 (6.3%)

a The values in parentheses give the percentage contribution to the total attractive interactions Δ*E*_elstat_ + Δ*E*_orb_.

b The values in parentheses give the percentage contribution to the total orbital interactions Δ*E*_orb_.

We also want to comment on the Δ*E*_orb(rest)_ term, which is comparatively large in the systems with L = CO, CS, NO^+^. It stems from the relaxation of the fragment orbitals with respect to the isolated species. The central N_2_ moieties in the latter complexes have significantly shorter N–N distances than in the ^1^Γ_g_ singlet state (1.606 Å at the M06-2X/cc-pVTZ level), and the electronic relaxation is, therefore, larger than in the complexes with L = N_2_, CN^−^ which have longer central N–N bonds (Fig. 1). The impact of the geometry relaxation on the BDE and the stability of molecules has been pointed out by Bickelhaupt in his activation strain model.^28^ The strong influence of the fragment relaxation and the geometry of the interacting species becomes obvious when the trend of the interaction energy Δ*E*_int_ between the frozen fragments (L = CS > CO > CN^−^ > NO^+^ > N_2_) is compared with the BDEs that are calculated using the fragments at their equilibrium geometries and electronic ground state (L = CS > CO > CN^−^ > N_2_ > NO^+^). Finally, we want to mention that the chemistry of N_2_(PPh_3_)_2_ was recently studied in joint experimental and theoretical works by the groups of Stephan and Grimme, which showed a surprising reactivity of the member of the N_2_L_2_ compound class.^29^

## Summary and conclusion

Geometry optimizations of the compounds N_2_L_2_ (L = N_2_, CO, CS, NO^+^, CN^−^) at the CCSD(T)/cc-pVTZ level give the energy minimum structures with a *trans*-periplanar arrangement of the L_2_ ligands at the N_2_ unit. Attempts to localize a *syn* isomer failed, and the calculations gave the *trans* isomer as the only conformational minimum. Energy calculations suggest that all compounds N_2_L_2_ (L = N_2_, CO, CS, NO^+^, CN^−^) may be synthesized and should be observable under appropriate conditions. The complexes with L = N_2_, CO, NO^+^, CN^−^ are predicted as thermodynamically unstable for dissociation into N_2_ + 2 L with Δ*G*^298^ value lying in between −257 kcal mol^−1^ (L = NO^+^) and −73 kcal mol^−1^ (L = CO), but the adduct N_2_(CS)_2_ is calculated as slightly stable with Δ*G*^298^ = 4 kcal mol^−1^. The homolytic dissociation reaction into two fragments N_2_L_2_ → 2 NL is energetically less favorable than the heterolytic fragmentation N_2_L_2_ → N_2_ + 2 L, which proceeds synchronously but asymmetrically. The activation barriers for the fragmentation reaction N_2_L_2_ → N_2_ + 2 L have values between Δ*G*^≠^(298 K) = 17 kcal mol^−1^ for L = N_2_ and Δ*G*^≠^(298 K) = 84 kcal mol^−1^ for L = CS. The calculated vibrational frequencies show that all molecules N_2_L_2_ have a characteristic vibrational mode with the second highest wavenumber *ν*_as_ that comes from the antisymmetric stretch of the ligands L, which is blue shifted for L = CO (Δ = 55 cm^−1^) and L = NO^+^ (Δ = 118 cm^−1^) but it is red shifted for L = CS (Δ = −54 cm^−1^) and L = CN^−^ (Δ = −133 cm^−1^) relative to the *ν*_as_ mode of L = N_2_.

The analysis of the bonding situation using the charge distribution reveals that there is a total charge donation L→(N_2_)←L in all complexes ranging between 1.38 *e* (L = CN^−^) and 0.56 *e* (L = N_2_), except in the dication with L = NO^+^, where a small backdonation in reverse direction L→(N_2_)←L with 0.10 *e* is calculated. EDA-NOCV calculations of N_6_ using the central N_2_ moiety and the terminal N_2_ ligands as interacting fragments in various electronic states and with different partial charges show that the best description of the bonding situation is given in terms of dative interactions N_2_→(N_2_)←N_2_ between central N_2_ in the excited (1)^1^Γ_g_ singlet state and the terminal N_2_ fragments in the ^1^Σ_g_^+^ electronic ground state. In contrast to the results for N_6_, the best description of the complexes with L = CO, CS, NO^+^ is calculated for the interactions between the central N_2_ in the ^5^Σ_u_^+^ quintet state and the terminal ligands in the symmetry-adapted (L)_2_ quintet state, which indicates electron-sharing double bonds between the fragments. For N_2_L_2_ with L = CN^−^, it is found that the bonding is best described for the interaction between N_2_^−^ in the electronic quartet (^4^Σ_u_^+^) state and the terminal (L)_2_^−^ ligand as symmetry-adapted quartet. A comparative analysis of the five N_2_L_2_ compounds using the same fragments with central N_2_ in the excited (1)^1^Γ_g_ singlet state and the terminal L_2_ fragments in the ^1^Σ_g_^+^ electronic ground state reveals that the *σ* donation L→(N_2_)←L makes the largest contribution to the stabilizing interactions and that the π backdonation L←(N_2_)←L is much weaker. In contrast to the common bonding model for N_6_ using Lewis structures N^−^=N^+^=N–N=N^+^=N^−^, the donor–acceptor model N_2_→(N_2_)←N_2_ explains that the lowest activation barrier is found for the concerted cleavage of the two formal double bonds, leading to the experimentally observed dissociation into 3 N_2_.

## Author contributions

G. F. and S. P. conceived the project, wrote the draft and finalized it, Y. L., C. D. and L. X. performed the calculations, Y. L. analyzed the data. All authors took part in the discussions and approved the final version.

## Conflicts of interest

The authors declare no conflict of interest.

## Supplementary Material

SC-017-D5SC08399K-s001

## Data Availability

The data supporting this article have been included as part of the supplementary information (SI). Supplementary information is available. See DOI: https://doi.org/10.1039/d5sc08399k.
