# Supplementary material for: Dinitrogen complexes N2L2 (L = N2, CO, CS, NO+, CN−)
Source: Chem Sci. 2026 Feb 9;17(14):7310–20. doi: 10.1039/d5sc08399k (PMC12919405; doi:10.1039/d5sc08399k)
Supplement: SC-017-D5SC08399K-s001 [file SC-017-D5SC08399K-s001.pdf]

Supplementary Information (SI) for Chemical Science.
This journal is © The Royal Society of Chemistry 2026

Supporting Information for

Dinitrogen Complexes N_2L_2 (L = N_2, CO, CS, NO^+, CN^-)

Yahui Li, Chengxiang Ding, Lianbin Xie, Sudip Pan, and Gernot Frenking

Tables S1 – S11

Figures S1 – S14

Table S1. Computed the electronic energies (ΔE) and zero-point energies corrected electronic energies (ΔE_0) of $\mathbf{N_2L_2}$ (L = N_2, CO, CS, CN^-, NO^+) at the M06-2X/cc-pVTZ level. All energetic values are given in kcal/mol.

$\mathbf{N_2L_2}$	Singlet	Triplet
	ΔE [ΔE_0]	ΔE [ΔE_0]
$N_2(N_2)_2$	0.0 [0.0]	48.8 [47.0]
$N_2(CO)_2$	0.0 [0.0]	67.7 [65.7]
$N_2(CS)_2$	0.0 [0.0]	51.5 [49.5]
$[N_2(CN)_2]^{2-}$	0.0 [0.0]	48.2 [46.1]
$[N_2(NO)_2]^{2+}$	0.0 [0.0]	42.8 [39.7]

Table S2. Computed bond dissociation energies De, zero-point energies corrected bond dissociation energies D_0, enthalpy change ΔH and free energy change ΔG of the processes (a) $N_2L_2 \rightarrow N_2 + 2L$ and (b) $N_2L_2 \rightarrow 2NL$ at the M06-2X/cc-pVTZ level level. All values are given in kcal/mol.

Complex	**D_e**	**D_0**	**ΔH**	**ΔG**
		**(a) $N_2L_2 \rightarrow N_2 + 2L$**		
$N_2(N_2)_2$	-172.9	-178.1	-175.8	-194.4
$N_2(CO)_2$	-42.2	-48.4	-46.3	-65.6
$N_2(CS)_2$	37.5	31.8	33.6	14.0
$[N_2(CN)_2]^{2-}$	-110.1	-115.6	-113.4	-133.1
$[N_2(NO)_2]^{2+}$	-231.6	-237.0	-234.7	-255.1
		**(b) $N_2L_2 \rightarrow 2NL$**		
$N_2(N_2)_2$	39.1	35.2	35.8	26.1
$N_2(CO)_2$	65.7	62.3	62.9	53.0
$N_2(CS)_2$	31.9	27.9	28.7	17.6
$[N_2(CN)_2]^{2-}$	-46.1	-51.6	-50.5	-56.7
$[N_2(NO)_2]^{2+}$	-69.6	-75.7	-74.4	-86.8

Table S3. The computed T_1 diagnostic of $N_2(L)_2$ complexes and TS for $N_2(L)_2 \rightarrow N_2 + 2L$ dissociation using the CCSD wave function.

Complexes	T_1
$N_2(CO)_2$	0.017
$N_2(N_2)_2$	0.018
$N_2(CS)_2$	0.016
$[N_2(NO)_2]^{2+}$	0.023
$[N_2(CN)_2]^{2-}$	0.015
$N_2(CO)_2^{‡}$	0.024
$N_2(N_2)_2^{‡}$	0.020
$N_2(CS)_2^{‡}$	0.022
$[N_2(NO)_2]^{2+‡}$	0.025
$[N_2(CN)_2]^{2-‡}$	0.020

Table S4. Computed IR frequencies and intensities (km mol^{-1}) at M06-2X / cc-pVTZ level for **N_2L_2. Notation:** ν (stretching), δ (bending) and ω (rocking kind of bending).

**$N_2(N_2)_2$**			
Assignment	Frequencies	Intensities	
ω_s**(N_2-N-N-N_2)**	**52.4**	**0.6**	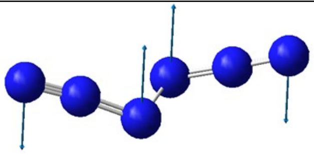
ω_{as}**(N_3-N_3)**	**171.3**	**2.4**	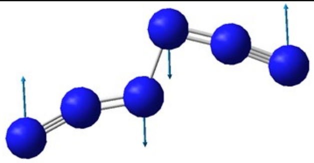
ω_s**(N_3-N_3)**	**298.2**	**0.0**	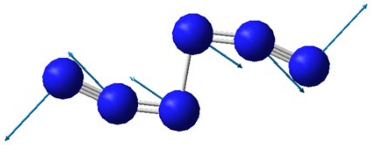
δ**s(N_2-NN)**	**549.9**	**16.5**	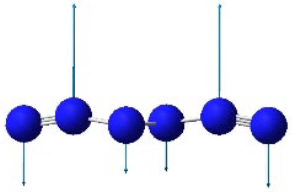
ω_s**(N_2-NN- N_2)**	**584.4**	**0.0**	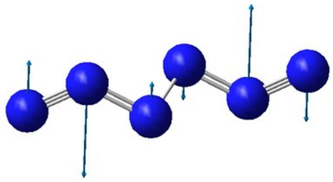
ω_s**(N_3-N_3)**	**644.7**	**0.0**	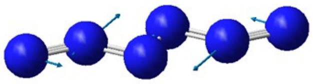
ω_{as} **(N_2-NN- N_2)**	**692.3**	**39.5**	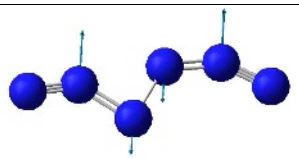
ω_s**(N_2-NN- N_2)**	**949.5**	**0.0**	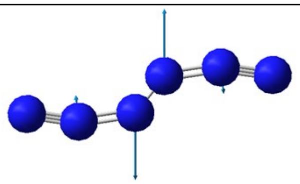

**νas(N_2-NN-N_2)**	**1275.0**	**198.4**	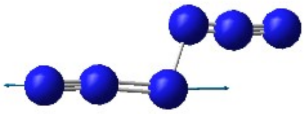
**νs(N_2-NN-N_2)**	**1346.8**	**C 0.0**	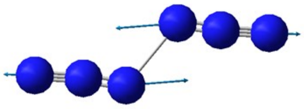
**ν_{as}(N-N-N)**	**2263.7**	**1514.3**	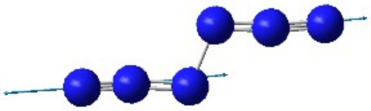
**ν_s(N-N-N)**	**2334.0**	**0.0**	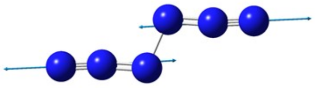
		**$N_2(CO)_2$**	
Assignment	Frequencies	Intensities	
**ω_s(OC-N-N-CO)**	**64.8**	**0.0**	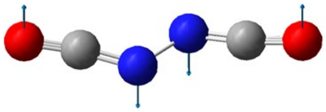
**ω_{as}(OCN-NCO)**	**121.5**	**11.1**	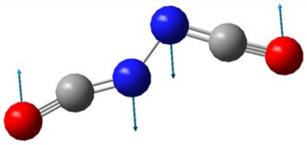
**ω_s(OCN-NCO)**	**2497**	**0.0**	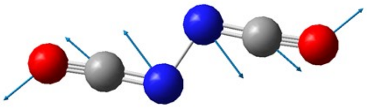
**δs(OC-NN)**	**577.2**	**0.0**	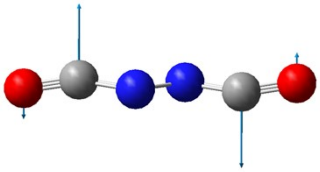
**ω_s(OC-NN-CO)**	**577.2**	**49.3**	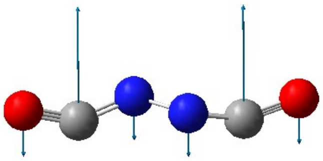
**ω_s(OCN-NCO)**	**653.9**	**0.0**	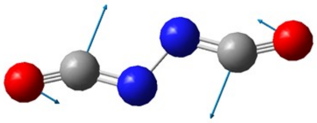

ω_{as} (OC-N-N-CO)	**688.5**	**97.9**	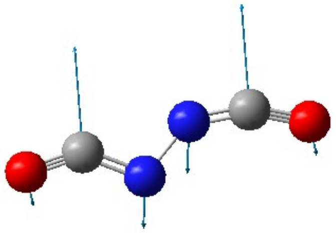
ω_s(OC-N-N-CO)	**870.9**	**0.0**	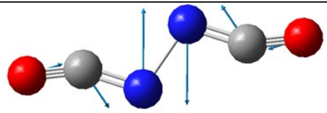
**νas(OC-NN-CO)**	**1362.4**	**0.8**	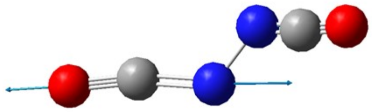
**νs(OC-NN-CO)**	**1566.5**	**0.0**	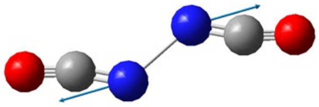
ν_{as}(O-C-N)	**2317.2**	**2628.1**	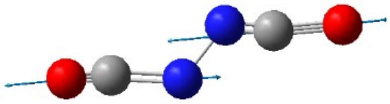
ν_s(O-C-N)	**2398.5**	**0.0**	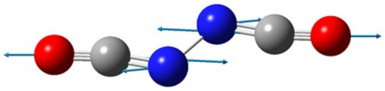
	$N_2(CS)_2$		
Assignment	Frequencies	Intensities	
ω_s(SC-N-N-CS)	**61.6**	**0.2**	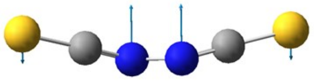
ω_{as}(SCN-NCS)	**76.4**	**16.4**	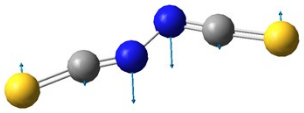
ω_s(SCN-NCS)	**183.3**	**0.0**	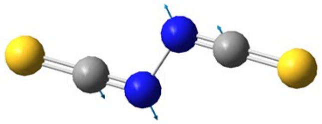
**δs(SC-NN)**	**429**	**0.0**	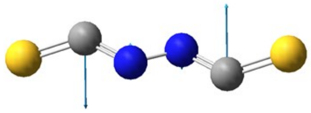
ω_s(SC-NN-CS)	**465.6**	**0.0**	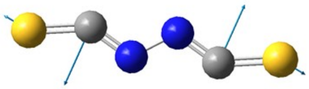

**ω_s(SCN-NCS)**	**465.7**	**2.6**	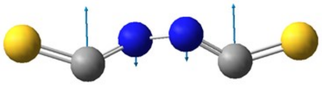
**ω_{as} (SC-N-N-CS)**	**525.1**	**226.1**	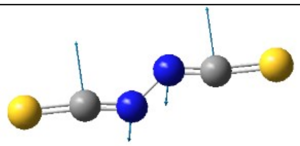
**ω_s(SC-N-N-CS)**	**666.7**	**0.0**	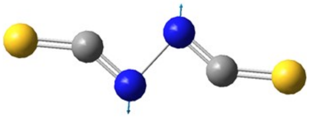
**νas(SC-NN-CS)**	**904.3**	**116.3**	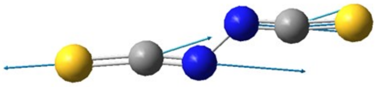
**νs(SC-NN-CS)**	**1278.2**	**0.0**	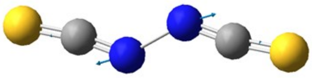
**νas(S-C-N)**	**2021.8**	**3314.4**	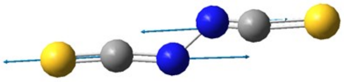
**νs(S-C-N)**	**2125.4**	**0.0**	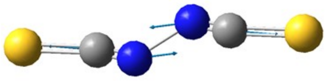
**$[N_2(NO)_2]^{2+}$**			
Assignment	Frequencies	Intensities	
**ω_s(ON-N-N-NO)**	**98.1**	**0.6**	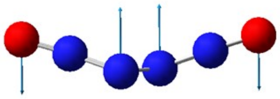
**ω_{as}(ONN-NNO)**	**165.1**	**5.7**	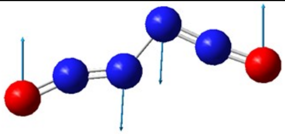
**ω_s(ONN-NNO)**	**315.3**	**0.0**	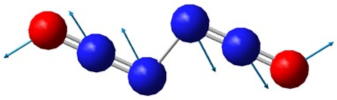
**δs(ON-NN)**	**534.8**	**16.7**	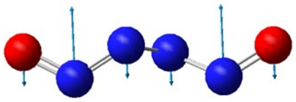
**ω_s(ON-NN-NO)**	**551.6**	**0.0**	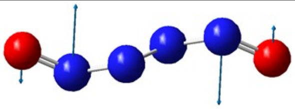

ω_s(ONN-NNO)	**635.1**	**0.0**	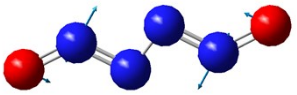
ω_{as} (ON-N-N-NO)	**663.1**	**64.9**	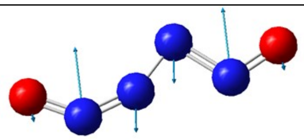
ω_s(ON-N-N-NO)	**877.6**	**0.0**	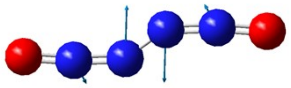
**νas(ON-NN-NO)**	**1427.8**	**50.7**	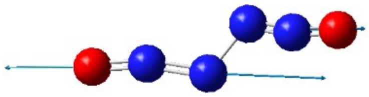
**νs(ON-NN-NO)**	**1557.5**	**0.0**	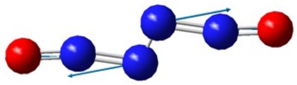
ν_{as}(O-N-N)	**2381.4**	**1865.2**	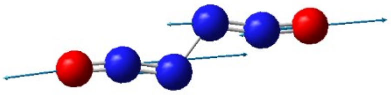
ν_s(O-N-N)	**2392.5**	**0.0**	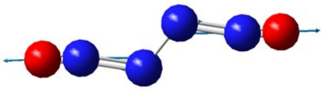
	**$[N_2(CN)_2]^{2-}$**		
Assignment	Frequencies	Intensities	
ω_s(NC-N-N-CN)	**44.5**	**3.5**	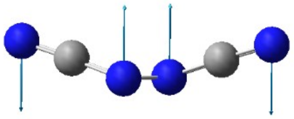
ω_{as}(NCN-NCN)	**157.3**	**16.4**	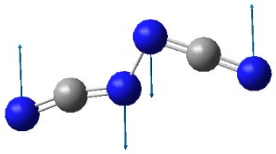
ω_s(NCN-NCN)	**291.3**	**0.0**	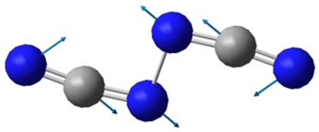
**δs(NC-NN)**	**602.6**	**24.2**	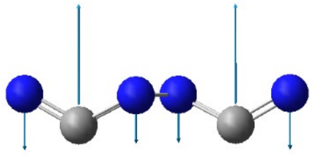

**ω_s(NC-NN-CN)**	**610.9**	**0.0**	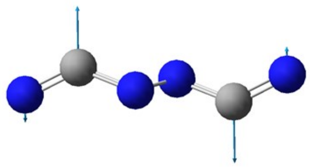
**ω_s(NCN-NCN)**	**629.6**	**0.0**	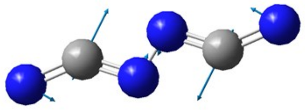
**ω_{as} (NC-N-N-CN)**	**710.3**	**45.8**	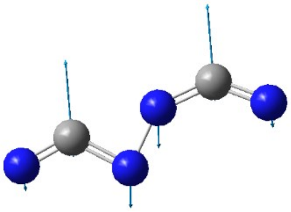
**ω_s(NC-N-N-CN)**	**869.2**	**0.0**	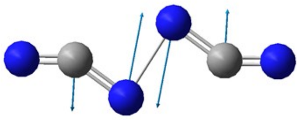
**νas(NC-NN-CN)**	**1219.8**	**51.9**	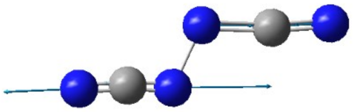
**νs(NC-NN-CN)**	**1316.8**	**0.0**	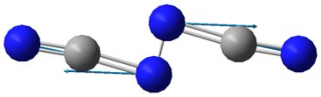
**νas(N-C-N)**	**2130.9**	**2195.7**	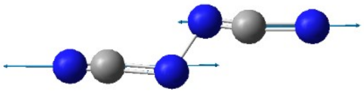
**νs(N-C-N)**	**2188.2**	**0.0**	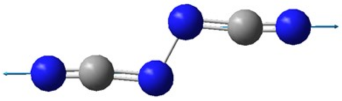

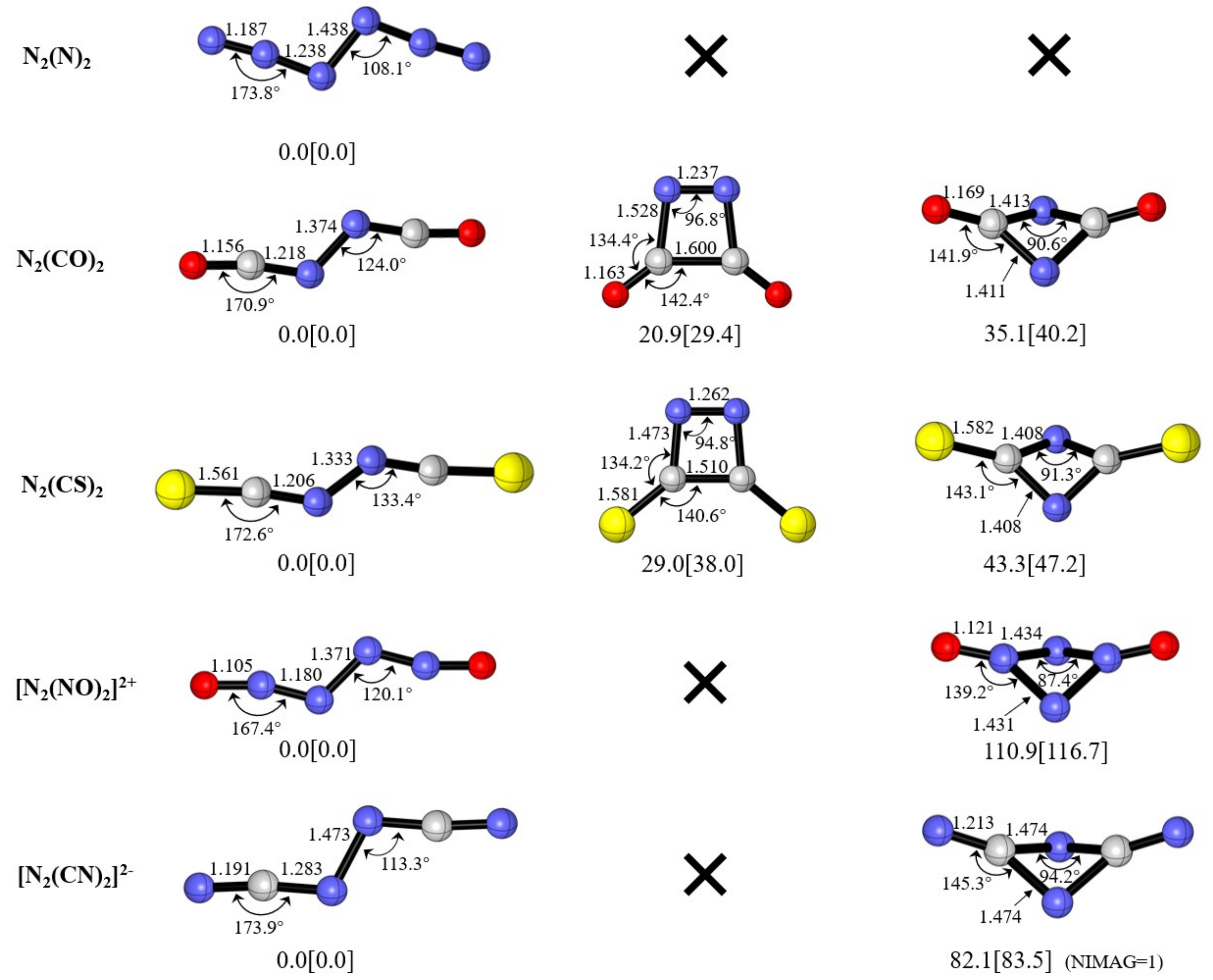

Figure S1. Calculated isomers of N_2L_2 complexes at the CCSD(T)/cc-pVTZ//M06-2X/cc-pVTZ level. The values in square brackets are at the M06-2X/cc-pVTZ level. The bond lengths are given in Å, bond angles in degree. Relative energies are in kcal/mol.

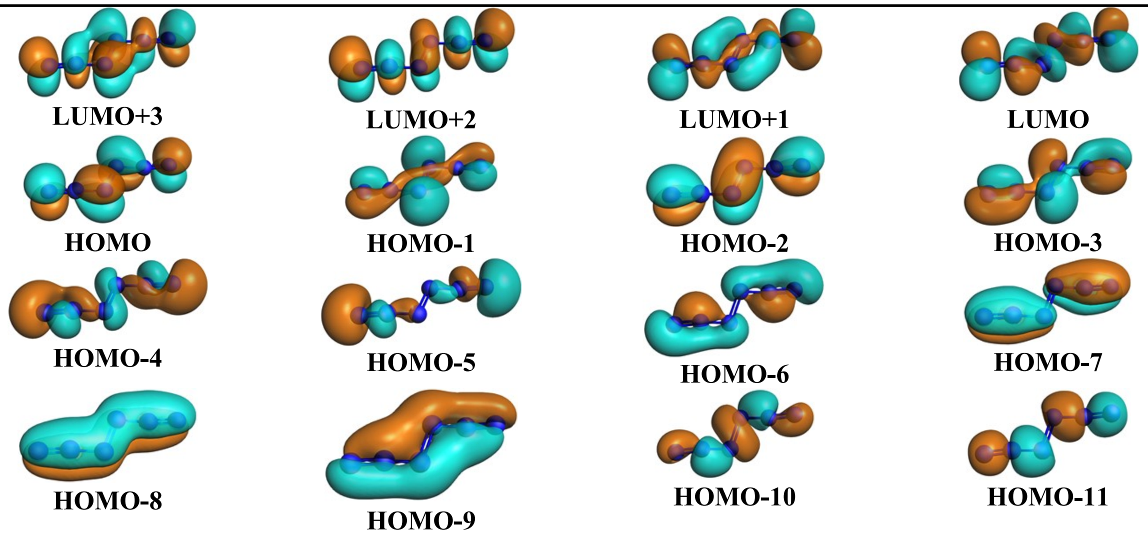

Figure S2. Plot of the most important MOs of $\mathbf{N_2(N_2)_2}$ at the CCSD(T)/cc-pVTZ level.

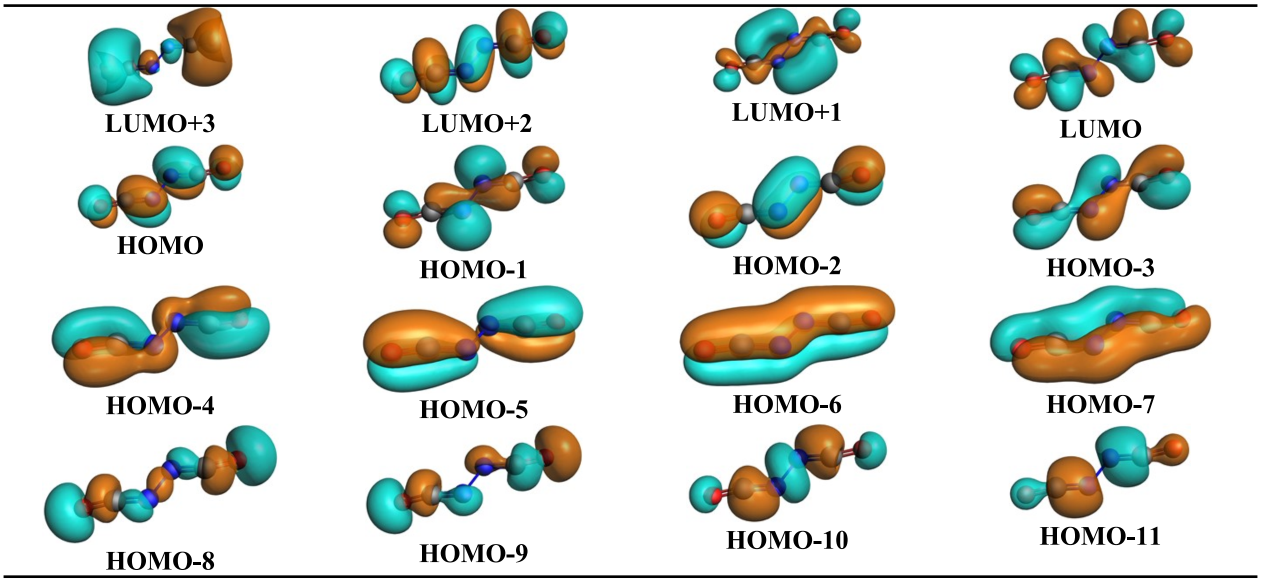

Figure S3. Plot of the most important MOs of $\mathbf{N_2(CO)_2}$ at the CCSD(T)/cc-pVTZ level.

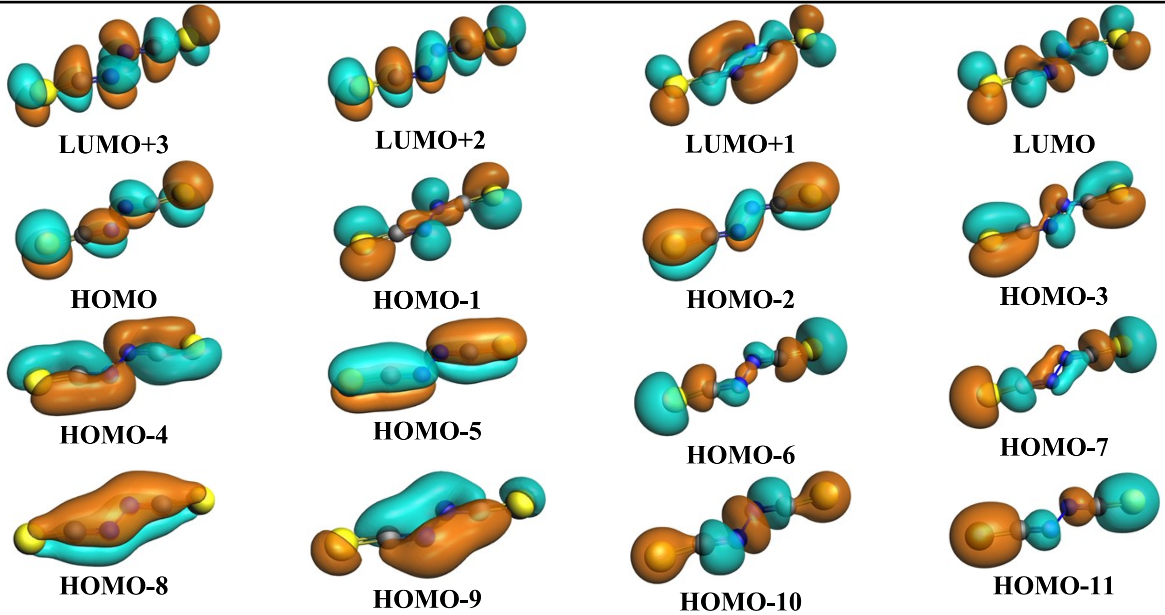

Figure S4. Plot of the most important MOs of $\mathbf{N_2(CS)_2}$ at the CCSD(T)/cc-pVTZ level.

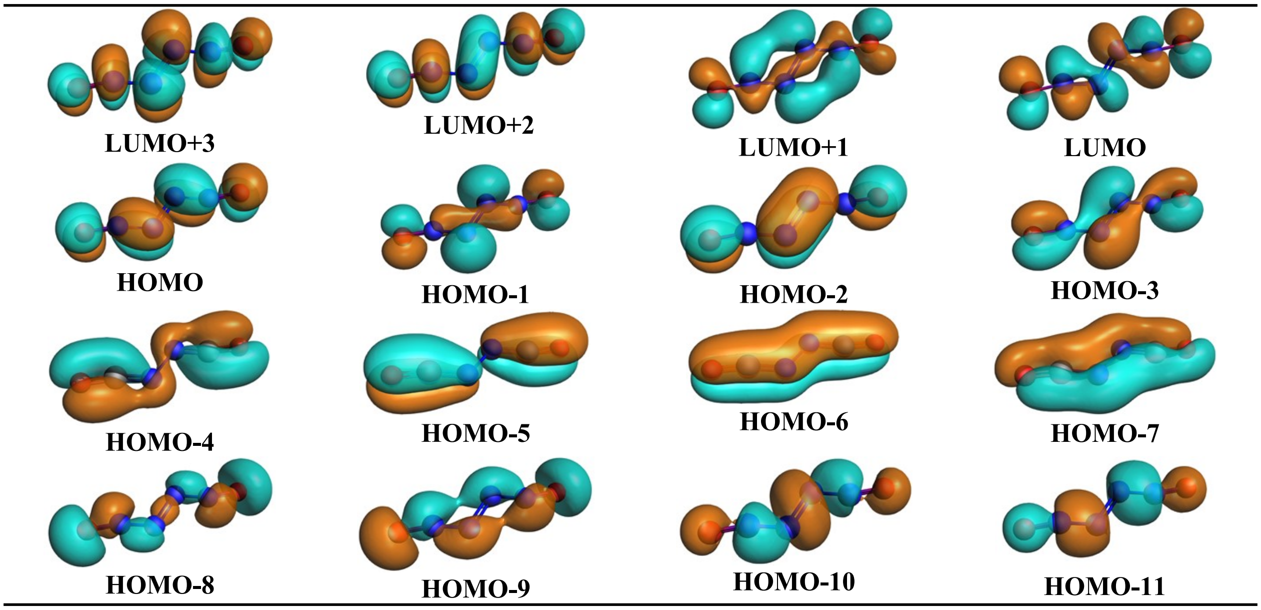

Figure S5. Plot of the most important MOs of **$[N_2(NO)_2]^{2+}$** at the CCSD(T)/cc-pVTZ level.

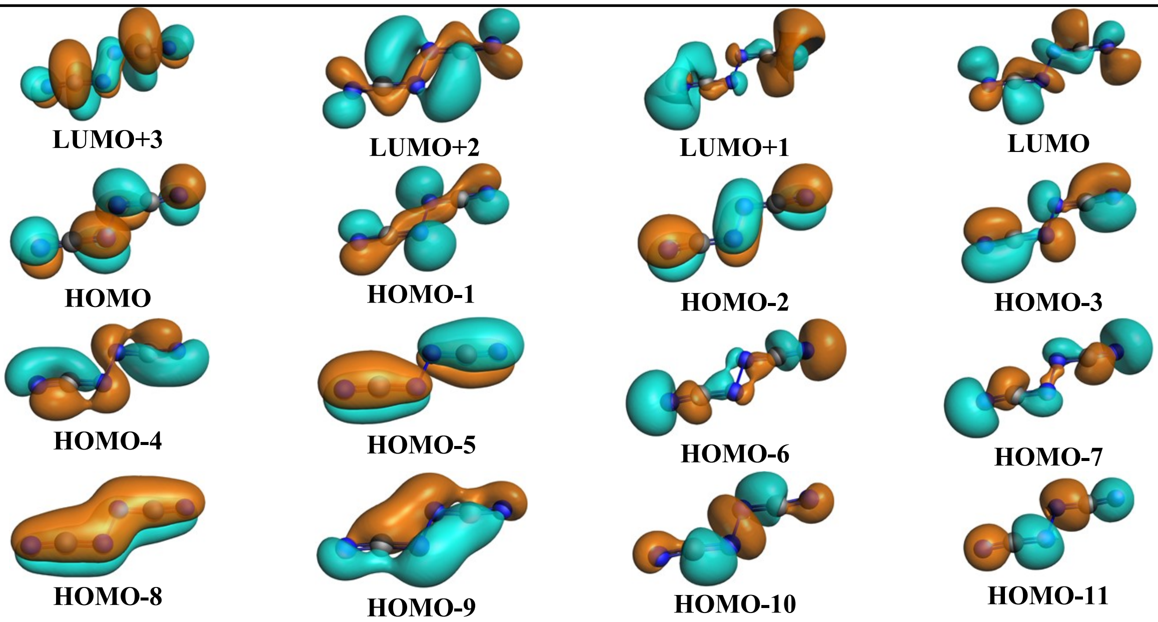

Figure S6. Plot of the most important MOs of **$[N_2(CN)_2]^{2-}$** at the CCSD(T)/cc-pVTZ level.

Table S5. EDA-NOCV results of complex **N_2L_2 (L=CO)** considering **L_2** as one fragment and the rest **N_2** as another at the M06-2X/TZ2P-ZORA//CCSD(T)/cc-pVTZ level. Energy values are given in kcal mol^{-1}.

Energies	Interaction	**NN** (Singlet) + **2L** (Singlet)	**NN** (Triplet) + **2L** (Triplet)	**NN** (Quintuplet) + **2L** (Quintuplet)	**NN^-**(Doublet) + **$2L^+$**(Doublet)	**NN^-**(Quartet) + **$2L^+$**(Quartet)
ΔE_{int}		-306.0	-327.6	-464.3	-478.9	-579.3
ΔE_{Pauli}		1400.4	1198.3	895.2	1212.5	937.2
$\Delta E_{elstat}{}^a$		-530.8 (31.1%)	-470.7 (30.8%)	-472.7 (33.7%)	-671.8 (39.7%)	-657.5 (43.4%)
$\Delta E_{orb}{}^a$		-1175.7 (68.9%)	-1055.2 (69.2%)	-931.7 (66.3%)	-1019.6 (60.3%)	-859.0 (56.6%)
$\Delta E_{orb(1)}{}^b$	**L-NN-L** σ-bond (+,-)	-483.0 (41.1%)	-473.4 (44.9%)	-299.8 (32.2%)	-399.7 (39.2%)	-293.5 (34.2%)
$\Delta E_{orb(2)}{}^b$	**L-NN-L** σ-bond (+,+)	-434.3 (37.4%)	-316.4 (30.0%)	-260.4 (27.9%)	-309.5 (30.4%)	-254.4 (29.6%)
$\Delta E_{orb(3)}{}^b$	**L-NN-L** π-bond (+,-)	-76.1 (6.5%)	-125.7 (11.9%)	-169.0 (18.1%)	-130.8 (12.8%)	-113.6 (13.2%)
$\Delta E_{orb(4)}{}^b$	**L-NN-L** π-bond (+,+)	-45.2 (3.8%)	-40.2 (3.8%)	-114.0 (12.2%)	-50.8 (5.0%)	-50.0 (5.8%)
$\Delta E_{orb(rest)}{}^b$		-137.1 (11.7%)	-99.5 (9.4%)	-88.5 (9.5%)	-128.8 (12.6%)	-147.5 (17.2%)

[a]The values in parentheses give the percentage contribution to the total attractive interactions ΔE_{elstat} + ΔE_{orb}.

[b]The values in parentheses give the percentage contribution to the total orbital interactions ΔE_{orb}.

	Deformation density	Orbital NN		Orbital 2L
$\Delta\rho_{(1)}$	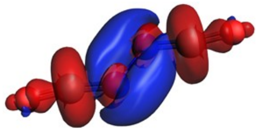 $\Delta E_{orb(1)}$ =-483.0 kcal/mol<br>$\lvert \nu_1 \rvert$ = 1.488	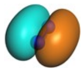 LUMO<br>ν= 1.450	←	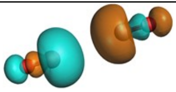 HOMO<br>ν= -1.179<br>+<br>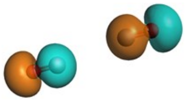 HOMO-3'<br>ν= -0.047
$\Delta\rho_{(2)}$	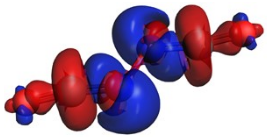 $\Delta E_{orb(2)}$ =-434.3 kcal/mol<br>$\lvert \nu_2 \rvert$ = 1.421	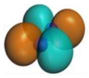 LUMO+1<br>ν= 1.178	←	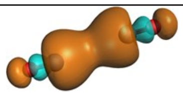 HOMO'<br>ν= -1.162<br>+<br>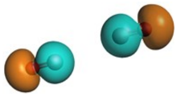 HOMO-3'<br>ν= -0.070
$\Delta\rho_{(3)}$	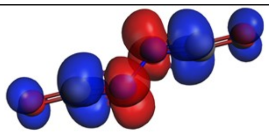 $\Delta E_{orb(3)}$ =-114.3 kcal/mol<br>$\lvert \nu_3 \rvert$ = 0.575	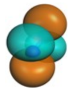 HOMO<br>ν= -0.761	→	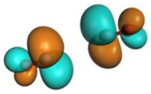 LUMO<br>ν=0.706
$\Delta\rho_{(4)}$	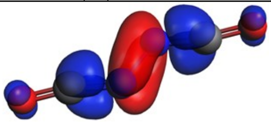 $\Delta E_{orb(4)}$ =-45.2 kcal/mol<br>$\lvert \nu_4 \rvert$ =0.512	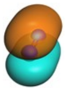 HOMO-1<br>ν= -0.302	→	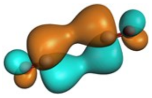 LUMO'<br>ν= 0.282

Figure S7. Plot of the deformation densities, $\Delta\rho_{(1\text{-}4)}$ shown as the electronic charge corresponding to $\Delta E_{orb(1\text{-}4)}$ and the related interacting orbitals of **N_2L_2 (L=CO)** at the M06-2X/TZ2P-ZORA//CCSD(T)/cc-pVTZ level using NN (singlet) + 2L (singlet) as interacting fragments. The eigenvalues ν indicate the size of the charge flow. The direction of charge flow is red → blue. The isovalue for $\Delta\rho_{(1\text{-}4)}$ is 0.003 au.

	Deformation density	Orbital		
		NN		2L
$\Delta\rho_{(1)}$	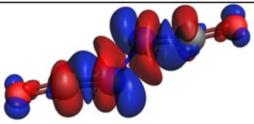<br>$\Delta E_{orb(1\alpha/1\beta)}$ =-293.5 kcal/mol<br>$\lvert\nu_{1\alpha/1\beta}\rvert$ = 0.337/0.695	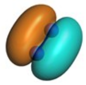<br>SOMO-1<br>$\nu_{1\alpha/1\beta}$=-0.246 /0.666	↔	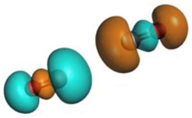<br>SOMO-1<br>$\nu_{1\alpha/1\beta}$=0.445/-0.421
$\Delta\rho_{(2)}$	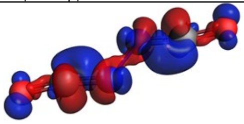<br>$\Delta E_{orb(2\alpha/2\beta)}$ =-254.4 kcal/mol<br>$\lvert\nu_{2\alpha/2\beta}\rvert$ = 0.413/0.641	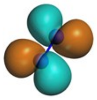<br>SOMO<br>$\nu_{2\alpha/2\beta}$=-0.402/0.524	↔	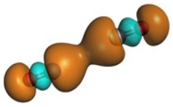<br>SOMO-1'<br>$\nu_{2\alpha/2\beta}$=0.173/-0.353
$\Delta\rho_{(3)}$	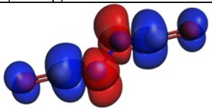<br>$\Delta E_{orb(3\alpha/3\beta)}$ =-113.6 kcal/mol<br>$\lvert\nu_{3\alpha/3\beta}\rvert$ = 0.479/0.665	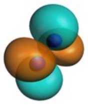<br>SOMO'<br>$\nu_{3\alpha/3\beta}$=-0.379/0.599	↔	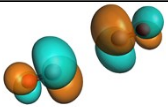<br>SOMO<br>$\nu_{3\alpha/3\beta}$=0.353/-0.611
$\Delta\rho_{(4)}$	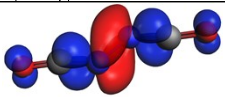<br>$\Delta E_{orb(4\alpha/4\beta)}$ =-50.0kcal/mol<br>$\lvert\nu_{4\alpha/4\beta}\rvert$ = 0.281/0.316	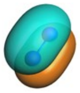<br>HOMO<br>$\nu_{4\alpha/4\beta}$=-0.151/-0.168	→	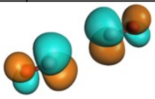<br>LUMO<br>$\nu_{4\alpha/4\beta}$=0.136/0.167

Figure S8. Plot of the deformation densities, $\Delta\rho_{(1-4)}$ shown as the sum of α and β electronic charge corresponding to $\Delta E_{orb(1-4)}$ and the related interacting orbitals of **N_2L_2 (L=CO)** at the M06-2X/TZ2P-ZORA//CCSD(T)/cc-pVTZ level using NN^- (Quartet) + $2L^+$ (Quartet) as interacting fragments. The eigenvalues ν indicate the size of the charge flow. The direction of charge flow is red → blue. The isovalue for $\Delta\rho_{(1)}$ is 0.003 au.

Table S6. EDA-NOCV results of complex $\mathbf{N_2L_2}$ **(L=CS)** considering $\mathbf{L_2}$ as one fragment and the rest $\mathbf{N_2}$ as another at the M06-2X/TZ2P-ZORA//CCSD(T)/cc-pVTZ level. Energy values are given in kcal mol^{-1}.

Energies	Interaction	**NN** (Singlet) + **2L** (Singlet)	**NN** (Triplet) + **2L** (Triplet)	**NN** (Quintuplet) + **2L** (Quintuplet)	**NN**$^-$(Doublet) + **2L**$^+$(Doublet)	**NN**$^-$(Quartet) + **2L**$^+$(Quartet)
ΔE_{int}		-387.0	-468.2	-422.3	-490.9	-666.2
ΔE_{Pauli}		1542.2	1347.0	1035.9	1367.7	1075.5
ΔE_{elstat}[a]		-595.4 (30.9%)	-540.0 (29.7%)	-475.9 (32.6%)	-696.6 (37.5%)	-676.6 (38.8%)
ΔE_{orb}[a]		-1333.8 (69.1%)	-1275.2 (70.3%)	-982.3 (67.4%)	-1162.0 (62.5%)	-1066.3 (61.2%)
$\Delta E_{orb(1)}$[b]	**L-NN-L** σ-bond (+,-)	-536.4 (40.2%)	-496.2 (38.9%)	-329.0 (33.5%)	-457.8 (39.4%)	-323.2 (30.3%)
$\Delta E_{orb(2)}$[b]	**L-NN-L** σ-bond (+,+)	-493.4 (37.0%)	-348.9 (27.4%)	-284.0 (28.9%)	-339.5 (29.2%)	-283.6 (26.6%)
$\Delta E_{orb(3)}$[b]	**L-NN-L** π-bond (+,-)	-134.6 (10.1%)	-153.3 (12.0%)	-150.3 (15.3%)	-159.0 (13.7%)	-149.3 (14.0%)
$\Delta E_{orb(4)}$[b]	**L-NN-L** π-bond (+,+)	-48.0 (3.6%)	-118.3 (9.3%)	-102.6 (10.4%)	-55.5 (4.8%)	-128.1 (12.0%)
$\Delta E_{orb(rest)}$[b]		-121.4 (9.1%)	-158.5 (12.4%)	-116.4 (11.8%)	-150.2 (12.9%)	-182.1 (17.1%)

[a]The values in parentheses give the percentage contribution to the total attractive interactions $\Delta E_{elstat} + \Delta E_{orb}$.

[b]The values in parentheses give the percentage contribution to the total orbital interactions ΔE_{orb}.

	Deformation density	Orbital		
		NN		2L
$\Delta\rho_{(1)}$	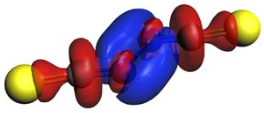<br>$\Delta E_{orb(1)}$ =-536.4 kcal/mol<br>$\lvert\nu_1\rvert$ = 1.557	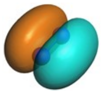<br>LUMO<br>ν= 1.499	←	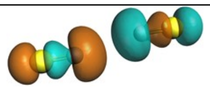<br>HOMO<br>ν= -1.180<br>+<br>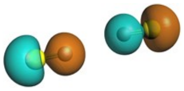<br>HOMO-3<br>ν= -0.100
$\Delta\rho_{(2)}$	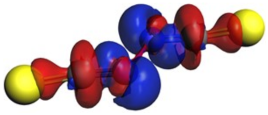<br>$\Delta E_{orb(2)}$ =-493.4 kcal/mol<br>$\lvert\nu_2\rvert$ = 1.524	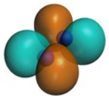<br>LUMO+1<br>ν= 1.161	←	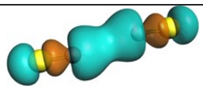<br>HOMO'<br>ν= -1.127<br>+<br>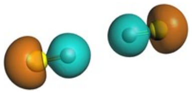<br>HOMO-3'<br>ν= -0.148
$\Delta\rho_{(3)}$	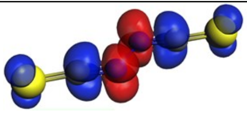<br>$\Delta E_{orb(3)}$ =-134.6 kcal/mol<br>$\lvert\nu_3\rvert$ = 1.114	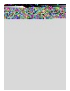<br>HOMO<br>ν= -0.897	→	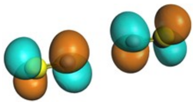<br>LUMO<br>ν=0.854
$\Delta\rho_{(4)}$	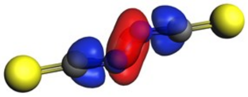<br>$\Delta E_{orb(4)}$ =-48.0 kcal/mol<br>$\lvert\nu_4\rvert$ = 0.621	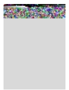<br>HOMO-1<br>ν= -0.321	→	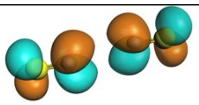<br>LUMO'<br>ν= 0.313

Figure S9. Plot of the deformation densities, $\Delta\rho_{(1\text{-}4)}$ shown as the electronic charge corresponding to $\Delta E_{orb(1\text{-}4)}$ and the related interacting orbitals of $\mathbf{N_2L_2}$ **(L=CS)** at the M06-2X/TZ2P-ZORA// CCSD(T)/cc-pVTZ level using NN (singlet) + 2L (singlet) as interacting fragments. The eigenvalues ν indicate the size of the charge flow. The direction of charge flow is red → blue. The isovalue for $\Delta\rho_{(1\text{-}4)}$ is 0.003 au.

	Deformation density	Orbital		
		NN		2L
$\Delta\rho_{(1)}$	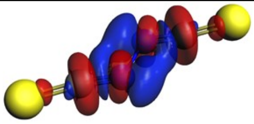 $\Delta E_{orb(1\alpha/1\beta)}$ =-329.0 kcal/mol $\lvert\nu_{1\alpha/1\beta}\rvert$ = 0.354/0.742	SOMO-1 $\nu_{1\alpha/1\beta}$=-0.203 /0.705	↔	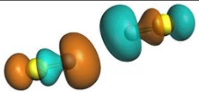 SOMO-1 $\nu_{1\alpha/1\beta}$=0.385/-0.474
$\Delta\rho_{(2)}$	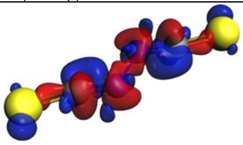 $\Delta E_{orb(2\alpha/2\beta)}$ =-284.0 kcal/mol $\lvert\nu_{2\alpha/2\beta}\rvert$ = 0.450/0.721	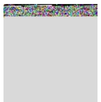 SOMO $\nu_{2\alpha/2\beta}$=-0.383/0.531	↔	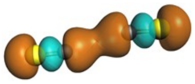 SOMO-1' $\nu_{2\alpha/2\beta}$=0.080/-0.354
$\Delta\rho_{(3)}$	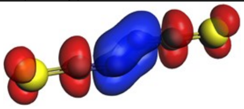 $\Delta E_{orb(3\alpha/3\beta)}$ =-150.3 kcal/mol $\lvert\nu_{3\alpha/3\beta}\rvert$ = 0.315/0.814	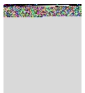 SOMO' $\nu_{3\alpha/3\beta}$=-0.165/0.807	↔	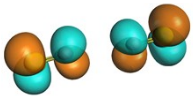 SOMO $\nu_{3\alpha/3\beta}$=0.144/-0.772
$\Delta\rho_{(4)}$	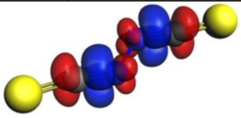 $\Delta E_{orb(4\alpha/4\beta)}$ =-102.6 kcal/mol $\lvert\nu_{4\alpha/4\beta}\rvert$ = 0.564/0.631	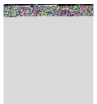 SOMO-1' $\nu_{4\alpha/4\beta}$=-0.454/0.415	↔	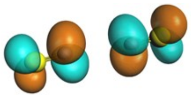 SOMO' $\nu_{4\alpha/4\beta}$=0.136/-0.496

Figure S10. Plot of the deformation densities, $\Delta\rho_{(1\text{-}4)}$ shown as the sum of α and β electronic charge corresponding to $\Delta E_{orb(1\text{-}4)}$ and the related interacting orbitals of $\mathbf{N_2L_2}$ **(L=CS)** at the M06-2X/TZ2P-ZORA//CCSD(T)/cc-pVTZ level using NN^- (Quintuplet) + $2L^+$ (Quintuplet) as interacting fragments. The eigenvalues ν indicate the size of the charge flow. The direction of charge flow is red → blue. The isovalue for $\Delta\rho_{(1)}$ is 0.003 au.

Table S7. EDA-NOCV results of complex $\mathbf{N_2L_2}$ **(L=NO⁺)** considering $\mathbf{L_2}$ as one fragment and the rest $\mathbf{N_2}$ as another at the M06-2X/TZ2P-ZORA//CCSD(T)/cc-pVTZ level. Energy values are given in kcal mol^{-1}.

Energies	Interaction	**NN** (Singlet) + **2L**$^{2+}$ (Singlet)	**NN** (Triplet) + **2L**$^{2+}$ (Triplet)	**NN** (Quintuplet) + **2L**$^{2+}$ (Quintuplet)	**NN**$^{-}$(Doublet) + **2L**$^{3+}$Doublet)	**NN**$^{-}$(Quartet) + **2L**$^{3+}$ (Quartet)
ΔE_{int}		-202.5	-411.1	-425.9	-812.3	-1071.5
ΔE_{Pauli}		1242.3	1108.3	929.8	1143.4	988.9
$\Delta E_{\text{elstat}}{}^{a}$		-356.9 (24.7%)	-351.7 (23.1%)	-368.8 (27.7%)	-810.0 (41.4%)	-872.8 (42.4%)
$\Delta E_{\text{orb}}{}^{a}$		-1088.0 (75.3%)	-1167.8 (76.9%)	-987.0 (72.8%)	-1145.7 (58.6%)	-1187.5 (57.6%)
$\Delta E_{\text{orb(1)}}{}^{b}$	**L-NN-L** σ-bond (+,-)	-373.2 (34.3%)	-336.0 (28.8%)	-310.2 (31.4%)	-344.7 (30.1%)	-320.3 (27.0%)
$\Delta E_{\text{orb(2)}}{}^{b}$	**L-NN-L** σ-bond (+,+)	-322.3 (29.6%)	-319.0 (27.3%)	-293.2 (29.7%)	-296.3 (25.9%)	-310.3 (26.1%)
$\Delta E_{\text{orb(3)}}{}^{b}$	**L-NN-L** π-bond (+,-)	-182.1 (16.7%)	-163.7 (14.0%)	-143.5 (14.5%)	-224.8 (19.6%)	-214.1 (18.0%)
$\Delta E_{\text{orb(4)}}{}^{b}$	**L-NN-L** π-bond (+,+)	-75.5 (6.9%)	-147.1 (12.6%)	-110.4 (11.2%)	-95.9 (8.3%)	-125.5 (10.6%)
$\Delta E_{\text{orb(rest)}}{}^{b}$		-134.9 (12.4%)	-202.0 (17.3%)	-129.7 (13.1%)	-184.0 (16.1%)	-217.3 (18.3%)

[a]The values in parentheses give the percentage contribution to the total attractive interactions $\Delta E_{\text{elstat}} + \Delta E_{\text{orb}}$.

[b]The values in parentheses give the percentage contribution to the total orbital interactions ΔE_{orb}.

	Deformation density	Orbital NN		Orbital 2L
$\Delta\rho_{(1)}$	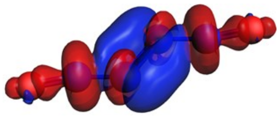 $\Delta E_{orb(1)}$ =-373.2 kcal/mol<br>$\lvert\nu_1\rvert$ = 1.308	LUMO<br>ν= 1.187	←	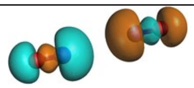 HOMO<br>ν= -0.796<br>+<br>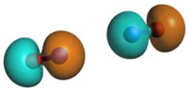 HOMO-3<br>ν= -0.153
$\Delta\rho_{(2)}$	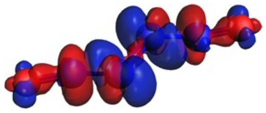 $\Delta E_{orb(2)}$ =-322.3 kcal/mol<br>$\lvert\nu_2\rvert$ = 1.174	LUMO+1<br>ν= 0.856	←	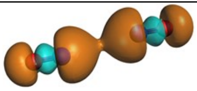 HOMO'<br>ν= -0.632<br>+<br>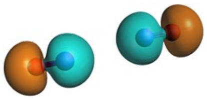 HOMO-3'<br>ν= -0.159
$\Delta\rho_{(3)}$	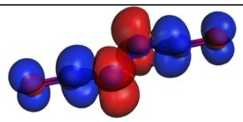 $\Delta E_{orb(3)}$ =-182.1 kcal/mol<br>$\lvert\nu_3\rvert$ = 1.214	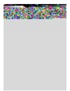 HOMO<br>ν= -0.993	→	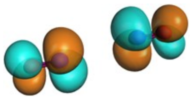 LUMO<br>ν=0.955
$\Delta\rho_{(4)}$	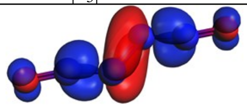 $\Delta E_{orb(4)}$ =-75.5 kcal/mol<br>$\lvert\nu_4\rvert$ = 0.740	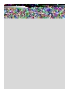 HOMO-1<br>ν= -0.437	→	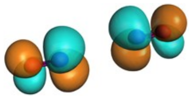 LUMO'<br>ν= 0.424

Figure S11. Plot of the deformation densities, $\Delta\rho_{(1\text{-}4)}$ shown as the electronic charge corresponding to $\Delta E_{orb(1\text{-}4)}$ and the related interacting orbitals of **N_2L_2 (L=NO^+)** at the M06-2X/TZ2P-ZORA// CCSD(T)/cc-pVTZ level using NN (singlet) + $2(L)^+$ (singlet) as interacting fragments. The eigenvalues ν indicate the size of the charge flow. The direction of charge flow is red → blue. The isovalue for $\Delta\rho_{(1\text{-}4)}$ is 0.003 au.

	Deformation density	Orbital		
		NN		2L
$\Delta\rho_{(1)}$	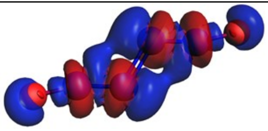 $\Delta E_{orb(1\alpha/1\beta)}$ =-310.2.0 kcal/mol $\lvert\nu_{1\alpha/1\beta}\rvert$ = 0.633/0.488	SOMO-1 $\nu_{1\alpha/1\beta}$=0.553 /-0.364	↔	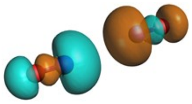 SOMO-1 $\nu_{1\alpha/1\beta}$=-0.246/0.565
$\Delta\rho_{(2)}$	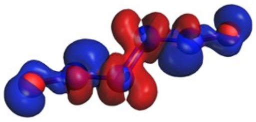 $\Delta E_{orb(2\alpha/2\beta)}$ =-293.2 kcal/mol $\lvert\nu_{2\alpha/2\beta}\rvert$ = 0.571/0.561	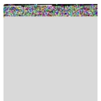 SOMO $\nu_{2\alpha/2\beta}$=0.384/-0.551	↔	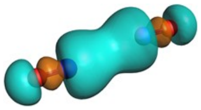 SOMO-1' $\nu_{2\alpha/2\beta}$=-0.175/0.481
$\Delta\rho_{(3)}$	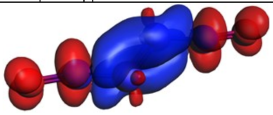 $\Delta E_{orb(3\alpha/3\beta)}$ =-143.5 kcal/mol $\lvert\nu_{3\alpha/3\beta}\rvert$ = 0.814/0.371	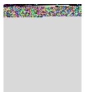 SOMO' $\nu_{3\alpha/3\beta}$=0.727/-0.218	↔	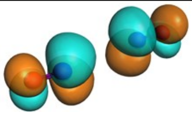 SOMO $\nu_{3\alpha/3\beta}$=-0.783/0.218
$\Delta\rho_{(4)}$	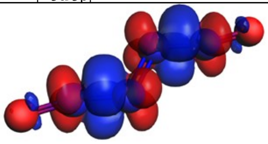 $\Delta E_{orb(4\alpha/4\beta)}$ =-110.4 kcal/mol $\lvert\nu_{4\alpha/4\beta}\rvert$ = 0.629/0.488	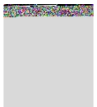 SOMO-1' $\nu_{4\alpha/4\beta}$=0.470/-0.498	↔	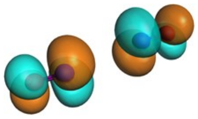 SOMO' $\nu_{4\alpha/4\beta}$=-0.510/0.480

Figure S12. Plot of the deformation densities, $\Delta\rho_{(1\text{-}4)}$ shown as the sum of α and β electronic charge corresponding to $\Delta E_{orb(1\text{-}4)}$ and the related interacting orbitals of **N_2L_2 (L= NO^+)** at the M06-2X/TZ2P-ZORA//CCSD(T)/cc-pVTZ level using NN (Quintuplet) + $2L^+$ (Quintuplet) as interacting fragments. The eigenvalues ν indicate the size of the charge flow. The direction of charge flow is red → blue. The isovalue for $\Delta\rho_{(1)}$ is 0.003 au.

Table S8. EDA-NOCV results of complex **N_2L_2 (L=CN^-)** considering **L_2** as one fragment and the rest **N_2** as another at the M06-2X/TZ2P-ZORA//CCSD(T)/cc-pVTZ level. Energy values are given in kcal mol^{-1}.

Energies	Interaction	**NN** (Singlet) + **2L^{2-}**Singlet)	**NN** (Triplet) + **2L^{2-}** (Triplet)	**NN** (Quintuplet) + **2L^{2-}** (Quintuplet)	**NN$^-$**(Doublet) + **2L$^-$**Doublet)	**NN$^-$**(Quartet) + **2L$^-$**Quartet)
ΔE_{int}		-301.4	-435.5	-460.5	-171.4	-375.5
ΔE_{Pauli}		1155.6	1013.2	773.0	1035.5	795.8
ΔE_{elstat}[a]		-484.9 (33.3%)	-453.2 (31.3%)	-406.2 (32.9%)	-373.3 (30.9%)	-361.4 (30.9%)
ΔE_{orb}[a]		-972.1 (66.7%)	-995.2 (68.7%)	-827.3 (67.1%)	-833.6 (69.1%)	-809.9 (69.1%)
$\Delta E_{orb(1)}$[b]	**L-NN-L** σ-bond (+,-)	-405.8 (41.7%)	-398.9 (40.1%)	-262.5 (31.7%)	-363.6 (43.6%)	-254.4 (31.4%)
$\Delta E_{orb(2)}$[b]	**L-NN-L** σ-bond (+,+)	-400.0 (41.1%)	-274.2 (27.6%)	-254.3 (30.7%)	-265.6 (31.9%)	-249.4 (30.8%)
$\Delta E_{orb(3)}$[b]	**L-NN-L** π-bond (+,-)	-70.4 (7.2%)	-155.2 (15.6%)	-151.9 (18.4%)	-78.2 (9.4%)	-134.7 (16.6%)
$\Delta E_{orb(4)}$[b]	**L-NN-L** π-bond (+,+)	-34.6 (3.6%)	-61.4 (6.2%)	-107.9 (13.0%)	-36.3 (4.4%)	-73.1 (9.0%)
$\Delta E_{orb(rest)}$[b]		-61.3 (6.3%)	-105.5 (10.6%)	-50.7 (6.1%)	-89.9 (10.8%)	-98.3 (12.1%)

[a]The values in parentheses give the percentage contribution to the total attractive interactions $\Delta E_{elstat} + \Delta E_{orb} + \Delta E_{disp}$.

[b]The values in parentheses give the percentage contribution to the total orbital interactions ΔE_{orb}.

	Deformation density	Orbital		
		NN		2L
$\Delta\rho_{(1)}$	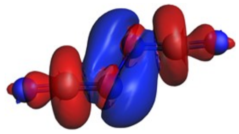 $\Delta E_{orb(1)}$ =-405.8 kcal/mol<br>$\lvert\nu_1\rvert$ = 1.350	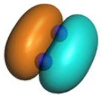 LUMO<br>ν= 1.391	←	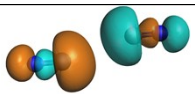 HOMO<br>ν= -1.120<br>+<br>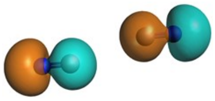 HOMO-2<br>ν= -0.122
$\Delta\rho_{(2)}$	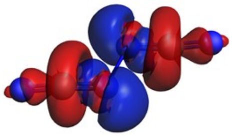 $\Delta E_{orb(2)}$ =-400.0 kcal/mol<br>$\lvert\nu_2\rvert$ = 1.423	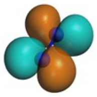 LUMO+1<br>ν= 1.267	←	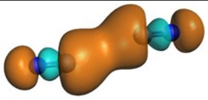 HOMO'<br>ν= -1.106<br>+<br>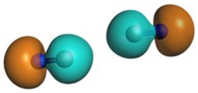 HOMO-2'<br>ν= -0.213
$\Delta\rho_{(3)}$	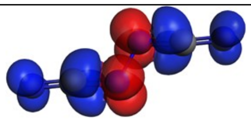 $\Delta E_{orb(3)}$ =-70.4 kcal/mol<br>$\lvert\nu_3\rvert$ = 0.860	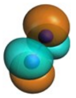 HOMO<br>ν= -0.569	→	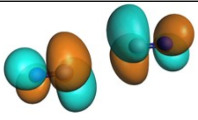 LUMO<br>ν=0.493
$\Delta\rho_{(4)}$	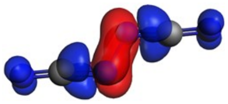 $\Delta E_{orb(4)}$ =-34.6 kcal/mol<br>$\lvert\nu_4\rvert$ = 0.534	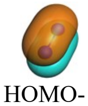 HOMO-1<br>ν= -0.255	→	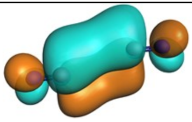 LUMO'<br>ν= 0.200

Figure S13. Plot of the deformation densities, $\Delta\rho_{(1\text{-}4)}$ shown as the electronic charge corresponding to $\Delta E_{orb(1\text{-}4)}$ and the related interacting orbitals of **N_2L_2 (L=CN^-)** at the M06-2X/TZ2P-ZORA//CCSD(T)/cc-pVTZ level using NN (singlet) + 2L (singlet) as interacting fragments. The eigenvalues ν indicate the size of the charge flow. The direction of charge flow is red → blue. The isovalue for $\Delta\rho_{(1\text{-}4)}$ is 0.003 au.

	Deformation density	Orbital		
		NN		2L
$\Delta\rho_{(1)}$	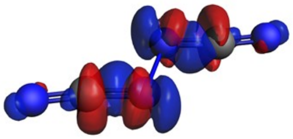 $\Delta E_{orb(1\alpha/1\beta)}$ =-254.4 kcal/mol $\lvert\nu_{1\alpha/1\beta}\rvert$ = 0.427/0.642	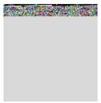 SOMO-1 $\nu_{1\alpha/1\beta}$=-0.375 /0.588	↔	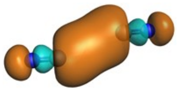 SOMO-1 $\nu_{1\alpha/1\beta}$=0.334/-0.373
$\Delta\rho_{(2)}$	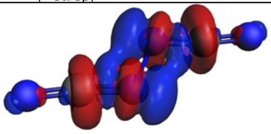 $\Delta E_{orb(2\alpha/2\beta)}$ =-249.4 kcal/mol $\lvert\nu_{2\alpha/2\beta}\rvert$ = 0.372/0.621	SOMO $\nu_{2\alpha/2\beta}$=-0.303/0.634	↔	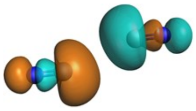 SOMO-1' $\nu_{2\alpha/2\beta}$=0.420/-0.317
$\Delta\rho_{(3)}$	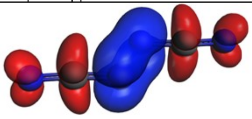 $\Delta E_{orb(3\alpha/3\beta)}$ =-134.7 kcal/mol $\lvert\nu_{3\alpha/3\beta}\rvert$ = 0.265/0.776	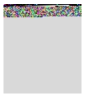 SOMO' $\nu_{3\alpha/3\beta}$=-0.126/0.853	↔	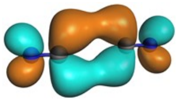 SOMO $\nu_{3\alpha/3\beta}$=0.09/-0.855
$\Delta\rho_{(4)}$	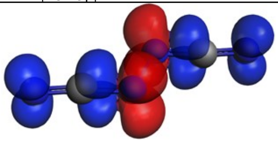 $\Delta E_{orb(4\alpha/4\beta)}$ =-73.1kcal/mol $\lvert\nu_{4\alpha/4\beta}\rvert$ = 0.428/0.428	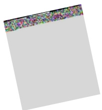 HOMO $\nu_{4\alpha/4\beta}$=-0.281/-0.287	→	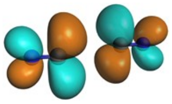 LUMO $\nu_{4\alpha/4\beta}$=0.256/0.278

Figure S14. Plot of the deformation densities, $\Delta\rho_{(1\text{-}4)}$ shown as the sum of α and β electronic charge corresponding to $\Delta E_{orb(1\text{-}4)}$ and the related interacting orbitals of **N_2L_2 (L= CN^-)** at the M06-2X/TZ2P-ZORA//CCSD(T)/cc-pVTZ level using NN^- (Quartet) + $2L^+$ (Quartet) as interacting fragments. The eigenvalues ν indicate the size of the charge flow. The direction of charge flow is red → blue. The isovalue for $\Delta\rho_{(1)}$ is 0.003 au.

Table S9. Coordinates of singlet $\mathbf{N_2L_2}$ compounds calculated at CCSD(T) cc-pVTZ level.

**$N_2(N_2)_2$**	E= -327.9142156 Hartree		
N	-0.395598000	0.607608000	0.000000000
N	0.395598000	-0.607608000	0.000000000
N	-0.395598000	-1.569160000	0.000000000
N	0.395598000	1.569160000	0.000000000
N	-1.001871000	-2.528018000	0.000000000
N	1.001871000	2.528018000	0.000000000
**$N_2(CO)_2$**	E= -335.6819974 Hartree		
N	-0.315331000	0.617014000	0.000000000
N	0.315331000	-0.617014000	0.000000000
C	-0.315331000	-1.670409000	0.000000000
C	0.315331000	1.670409000	0.000000000
O	-0.729273000	-2.758835000	0.000000000
O	0.729273000	2.758835000	0.000000000
**$N_2(CS)_2$**	E= -980.9204941 Hartree		
N	-0.278551000	0.617739000	0.000000000
N	0.278551000	-0.617739000	0.000000000
C	-0.278551000	-1.702593000	0.000000000
C	0.278551000	1.702593000	0.000000000
S	-0.788427000	-3.183514000	0.000000000
S	0.788427000	3.183514000	0.000000000
**$[N_2(NO)_2]^{2+}$**	E= -367.8472101 Hartree		
N	-0.315331000	0.617014000	0.000000000
N	0.315331000	-0.617014000	0.000000000
C	-0.315331000	-1.670409000	0.000000000
C	0.315331000	1.670409000	0.000000000
O	-0.729273000	-2.758835000	0.000000000
O	0.729273000	2.758835000	0.000000000
**$[N_2(CN)_2]^{2-}$**	E= -294.6524902 Hartree		
N	-0.384144000	0.636985000	0.000000000
N	0.384144000	-0.636985000	0.000000000
C	-0.384144000	-1.672029000	0.000000000
C	0.384144000	1.672029000	0.000000000
N	-0.984216000	-2.713049000	0.000000000
N	0.984216000	2.713049000	0.000000000

Table S10. Coordinates of transition states of $\mathbf{N_2L_2}$ compounds calculated at M062X cc-pVTZ level.

$N_2(N_2)_2$ ‡ E= -328.290010 Hartree

N	-1.602462000	-0.191493000	0.060886000
N	0.602568000	0.723441000	0.413736000
N	1.567410000	-0.087059000	0.051183000
N	-2.653146000	-0.508513000	0.049880000
N	2.496533000	-0.663589000	-0.115625000
N	-0.410903000	0.727213000	-0.460060000

$N_2(CO)_2$‡ E= -335.990255 Hartree

N	0.654377000	0.434181000	0.223835000
N	-0.478304000	0.746282000	-0.104805000
C	1.761246000	-0.085213000	-0.205314000
C	-1.842050000	-0.418255000	0.105144000
O	2.852836000	-0.402176000	0.027406000
O	-2.946297000	-0.253129000	-0.056429000

$N_2(CS)_2$ ‡ E= -981.864668 Hartree

N	-0.723673000	0.647916000	0.000385000
N	0.397903000	1.043783000	-0.000046000
C	-1.848664000	0.192636000	-0.000118000
C	1.913335000	-0.460944000	0.001653000
S	-3.306828000	-0.397815000	-0.000199000
S	3.425100000	-0.241687000	-0.000525000

$[N_2(NO)_2]^{2+}$ ‡ E= -368.204124 Hartree

N	0.537425000	-0.129170000	0.304152000
N	-0.490583000	0.523214000	0.008961000
N	1.695622000	-0.018474000	-0.168140000
N	-1.733938000	-0.222191000	-0.139149000
O	2.787512000	-0.071557000	-0.020613000
O	-2.794972000	-0.062650000	0.015517000

$[N_2(CN)_2]^{2-}$ ‡ E= -294.972598 Hartree

N	0.767524000	0.730935000	0.401635000
N	-0.286898000	0.851306000	-0.418006000
C	1.736188000	-0.108791000	0.056485000
C	-1.892025000	-0.256017000	0.087676000
N	2.689009000	-0.772827000	-0.121486000
N	-3.036060000	-0.496721000	0.014290000

Table S11. Coordinates of singlet $\mathbf{N_2L_2}$ isomer compounds calculated at CCSD(T)/cc-pVTZ//M06-2X/cc-pVTZ level.

$N_2(N_2)_2$ E= -328.3232337 Hartree

N	0.451962000	0.559397000	0.000000000
N	-0.451958000	-0.559380000	0.000000000
N	-1.608659000	-0.118168000	-0.000002000
N	1.608665000	0.118191000	0.000002000
N	-2.691052000	0.164575000	-0.000003000
N	2.691042000	-0.164615000	0.000003000

$N_2(CO)_2$ E= -336.1034887 Hartree

N	0.536507000	-0.428777000	0.000000000
N	-0.536506000	0.428777000	0.000000000
C	-1.699565000	0.065733000	0.000002000
C	1.699564000	-0.065729000	-0.000002000
O	-2.843308000	-0.100262000	0.000004000
O	2.843308000	0.100259000	-0.000004000

$N_2(CS)_2$ E= -982.0132947 Hartree

N	0.554225000	-0.370234000	0.000000000
N	-0.554225000	0.370232000	0.000000000
C	-1.730314000	0.101441000	0.000002000
C	1.730315000	-0.101445000	-0.000002000
S	-3.283723000	-0.047383000	0.000004000
S	3.283723000	0.047385000	-0.000004000

$[N_2(NO)_2]^{2+}$ E= -368.2528976 Hartree

N	-0.316220000	0.608265000	0.000000000
N	0.316220000	-0.608265000	0.000000000
O	-0.690279000	-2.644174000	0.000000000
O	0.690279000	2.644174000	0.000000000
N	-0.316220000	-1.604875000	0.000000000
N	0.316220000	1.604875000	0.000000000

$[N_2(CN)_2]^{2-}$ E= -295.0575226 Hartree

N	-0.376744000	0.632752000	0.000000000
N	0.376744000	-0.632752000	0.000000000
C	-0.376744000	-1.670768000	0.000000000
C	0.376744000	1.670768000	0.000000000
N	-0.970452000	-2.703053000	0.000000000
N	0.970452000	2.703053000	0.000000000

$N_2(CO)_2$ **isomer 1** E= -336.0566058 Hartree

C	0.799870000	-0.235742000	-0.000033000
N	-0.618614000	1.281448000	0.000016000
N	0.618610000	1.281449000	0.000209000
C	-0.799869000	-0.235744000	-0.000014000
O	-1.721539000	-0.944461000	-0.000098000
O	1.721542000	-0.944459000	-0.000064000

$N_2(CO)_2$ **isomer 2** E= -336.039365 Hartree

C	-1.004172000	-0.000695000	0.007999000
N	-0.000181000	-0.864914000	-0.477522000
C	1.004179000	0.000022000	0.007775000
N	0.000322000	0.866918000	-0.475736000
O	-2.101542000	-0.000405000	0.411068000
O	2.101413000	-0.000844000	0.411202000

$N_2(CS)_2$ **isomer 1** E= -981.9528074 Hartree

C	0.754903000	0.199443000	0.000037000
N	-0.630915000	1.666860000	0.000111000
N	0.630909000	1.666860000	0.000166000
C	-0.754902000	0.199443000	-0.000023000
S	-1.976346000	-0.804044000	-0.000192000
S	1.976348000	-0.804041000	0.000066000

$N_2(CS)_2$ **isomer 2** E= -981.9381286 Hartree

C	1.007217000	0.205030000	0.000536000
N	0.000397000	0.715112000	-0.841367000
S	-2.472798000	-0.390002000	0.000248000
S	2.472791000	-0.390038000	0.000220000
C	-1.007162000	0.204787000	-0.000448000
N	-0.000428000	0.716566000	0.840220000

$[N_2(NO)_2]^{2+}$ **isomer 2** E= -368.0668535 Hartree

N	0.001638000	-0.909365000	0.440520000
N	-0.001609000	0.910916000	0.439257000
N	0.989474000	-0.001173000	-0.054547000
N	-0.989555000	0.000977000	-0.054517000
O	2.074254000	0.000479000	-0.337181000
O	-2.074208000	-0.001665000	-0.337193000

$[N_2(CN)_2]^{2-}$ **isomer 2** E= -294.9243789 Hartree

C	-1.079110000	0.000264000	-0.040165000
N	0.000243000	-0.829905000	0.524521000
C	1.079145000	-0.000135000	-0.040133000
N	-0.000281000	0.829109000	0.525436000
N	2.205525000	0.000525000	-0.490566000
N	-2.205517000	0.000161000	-0.490564000
